# Integrating Computational Methods to Investigate the Macroecology of Microbiomes

**DOI:** 10.3389/fgene.2019.01344

**Published:** 2020-01-17

**Authors:** Rilquer Mascarenhas, Flávia M. Ruziska, Eduardo Freitas Moreira, Amanda B. Campos, Miguel Loiola, Kaike Reis, Amaro E. Trindade-Silva, Felipe A. S. Barbosa, Lucas Salles, Rafael Menezes, Rafael Veiga, Felipe H. Coutinho, Bas E. Dutilh, Paulo R. Guimarães, Ana Paula A. Assis, Anderson Ara, José G. V. Miranda, Roberto F. S. Andrade, Bruno Vilela, Pedro Milet Meirelles

**Affiliations:** ^1^ Institute of Biology, Federal University of Bahia, Salvador, Brazil; ^2^ Chemical Engineering Department, Polytechnic School of Federal University of Bahia, Salvador, Brazil; ^3^ Department of Ecology, Biosciences Institute, University of Sao Paulo, Sao Paulo, Brazil; ^4^ Institute of Geology, Federal University of Bahia, Salvador, Brazil; ^5^ Institute of Physics, Federal University of Bahia, Salvador, Brazil; ^6^ Center of Data and Knowledge Integration for Health (CIDACS), Instituto Gonçalo Muniz, Fundação Oswaldo Cruz, Brazil; ^7^ Evolutionary Genomics Group, Departamento de Producción Vegetal y Microbiología, Universidad Miguel Hernández de Elche, San Juan de Alicante, Spain; ^8^ Theoretical Biology and Bioinformatics, Utrecht University, Utrecht, Netherlands; ^9^ Centre for Molecular and Biomolecular Informatics, Radboud University Medical Centre, Nijmegen, Netherlands; ^10^ Department of Ecology, Biosciences Institute, University of Sao Paulo, Butantã, Brazil; ^11^ Institute of Mathematics, Federal University of Bahia, Salvador, Brazil

**Keywords:** microbial community modeling, microbial macroecology, spatial scales, machine learning, co-occurrence networks

## Abstract

Studies in microbiology have long been mostly restricted to small spatial scales. However, recent technological advances, such as new sequencing methodologies, have ushered an era of large-scale sequencing of environmental DNA data from multiple biomes worldwide. These global datasets can now be used to explore long standing questions of microbial ecology. New methodological approaches and concepts are being developed to study such large-scale patterns in microbial communities, resulting in new perspectives that represent a significant advances for both microbiology and macroecology. Here, we identify and review important conceptual, computational, and methodological challenges and opportunities in microbial macroecology. Specifically, we discuss the challenges of handling and analyzing large amounts of microbiome data to understand taxa distribution and co-occurrence patterns. We also discuss approaches for modeling microbial communities based on environmental data, including information on biological interactions to make full use of available Big Data. Finally, we summarize the methods presented in a general approach aimed to aid microbiologists in addressing fundamental questions in microbial macroecology, including classical propositions (such as “everything is everywhere, but the environment selects”) as well as applied ecological problems, such as those posed by human induced global environmental changes.

## Introduction

The purpose of macroecology is to describe spatial patterns of species distribution and abundance, as well as the mechanisms underlying such patterns (McGill, 2003; McGill and Nekola, 2010). The availability of large amounts of data (Hampton et al., 2013) has helped to uncover global ecological patterns in species distribution and abundance, greatly advancing the field of macroecology. This is highlighted by several studies discussing the contribution of microbial community investigations to a unified macroecological theory (Barberán et al., 2014; Blaser et al., 2016; Nelson et al., 2016; Shade et al., 2018). Strong evidence suggests that micro-organisms in deep display biogeographical patterns which are driven by dispersal processes, climate and evolutionary history, such as species-area and distance-decay associations (Horner-Devine et al., 2004; Astorga et al., 2012; Barberán et al., 2015). The field of microbial macroecology has therefore emerged as a promising research path to the unified understanding of ecological processes shaping patterns across different branches in the tree of life.

The contributions of microbiology to macroecology are currently possible largely due to the methodological advances in theoretical and computational tools for investigating microbiomes. Advances in molecular biology and DNA sequencing in the last decade have provided microbial ecologists with new tools allowing the extraction of an unprecedented amount of information from myriads of microbial communities (Snyder et al., 2009). As a result of the growing amount of stored data, new software has been developed for the systematic study of microbial communities on a macroecological scale. Integration among these tools, however, is not a simple task. Microbial macroecology stands to benefit from a formal summary describing the coupling of microbial community characteristics with spatial environmental information.

In this review, we summarize important conceptual challenges as well as computational and methodological opportunities in the study of microbial macroecology, in order to facilitate data integration. We begin by reviewing what has already been described in this field, specifically addressing the conceptual issues of transitioning from micro- to macro- scales when using micro-organisms as model systems. Then, we provide a comprehensive summary of computational tools for describing microbial communities and predicting the distribution of taxa across large spatial scales. Finally, we conclude by proposing a general framework to aid microbiologists in incorporating the peculiarities of micro-organisms into conceptual frameworks of macroecology, in order to promote the unification of microbial ecology and general ecology.

### What Have We Done So Far: A Brief Review of Macroecological Studies in Microbiology

Most macroecological studies of microbial communities to date sought primarily to describe patterns in large spatial scales, investigating whether biogeographical patterns exist for the microbiota (Noguez et al., 2005). Most studies were conducted in soil and marine environments and revealed that such patterns do exist. They suggest that environmental predictors for microbiomes could differ from those usually assumed for macroorganisms (i.e., temperature, precipitation and altitude; Fierer and Jackson, 2006); environmental features such as pH, edaphic conditions and land usage are stronger and better predictors for soil microbiomes. However, soil moisture and temperature also appear to be important to predict microbial community composition in some cases (Fierer and Jackson, 2006; Lauber et al., 2009; Drenovsky et al., 2010; Zhou et al., 2016). In marine environments, spatial structure for microbial communities appears to be less prominent (i.e., lower beta-diversity) in comparison to terrestrial and freshwater systems, which is probably due to the more homogeneous abiotic structure of the open ocean (Soininen, 2012) in relation to other environments. Additionally, temperature was a strong predictor for a latitudinal gradient pattern found in planktonic bacteria, with little importance from other variables, such as productivity and salinity (Fuhrman et al., 2008). One study suggested the influence of altitude—a factor that influences that altitudinal patterns of macroorganisms (Lomolino, 2001)—seem to be not relevant for micro-organisms (Fierer et al., 2011). By contrast, Delgado-Baquerizo et al. (2016) stated that altitude gradients are important drivers for microbial diversity considering a wide spatial range (0–4600 m). Finally, it was suggested that micro-organisms in the atmosphere follow a precipitation gradient at continental scales (Barberán et al., 2015). These studies show that some macroecological patterns exist at microbial scales and that they may be similar to those found for macroorganisms in some cases, but not similar in other instances. This raises the question: to which extent are these patterns ubiquitous through all domains of life?

Although much effort has been made to unravel microbial macroecological patterns, so far there is no consensus on which abiotic factors are good predictors of microbial community composition, hampering the implementation of macroecological models to microbial data. Additionally, even though the studies above show strong correlations between variables and microbiome composition, it is still unknown whether the used variables are true drivers of the observed processes, or whether they are actually correlated to unmeasured, confounding factors (Rahbek, 2005). Biotic interactions seem to be equally important in determining community composition; a modeling approach using Artificial Neural Network (Larsen et al., 2012) highlighted the importance of such interactions for creating more accurate models, and a recent study using large microbial community datasets suggested that rarer taxa are better predictors of community structure than environmental factors (Ramirez et al., 2018). Therefore, a modeling framework based on the conceptual idiosyncrasies of microbiomes is required.

### Conceptual Challenges for Transitioning Across Spatial and Temporal Scales

**Figure 1:**
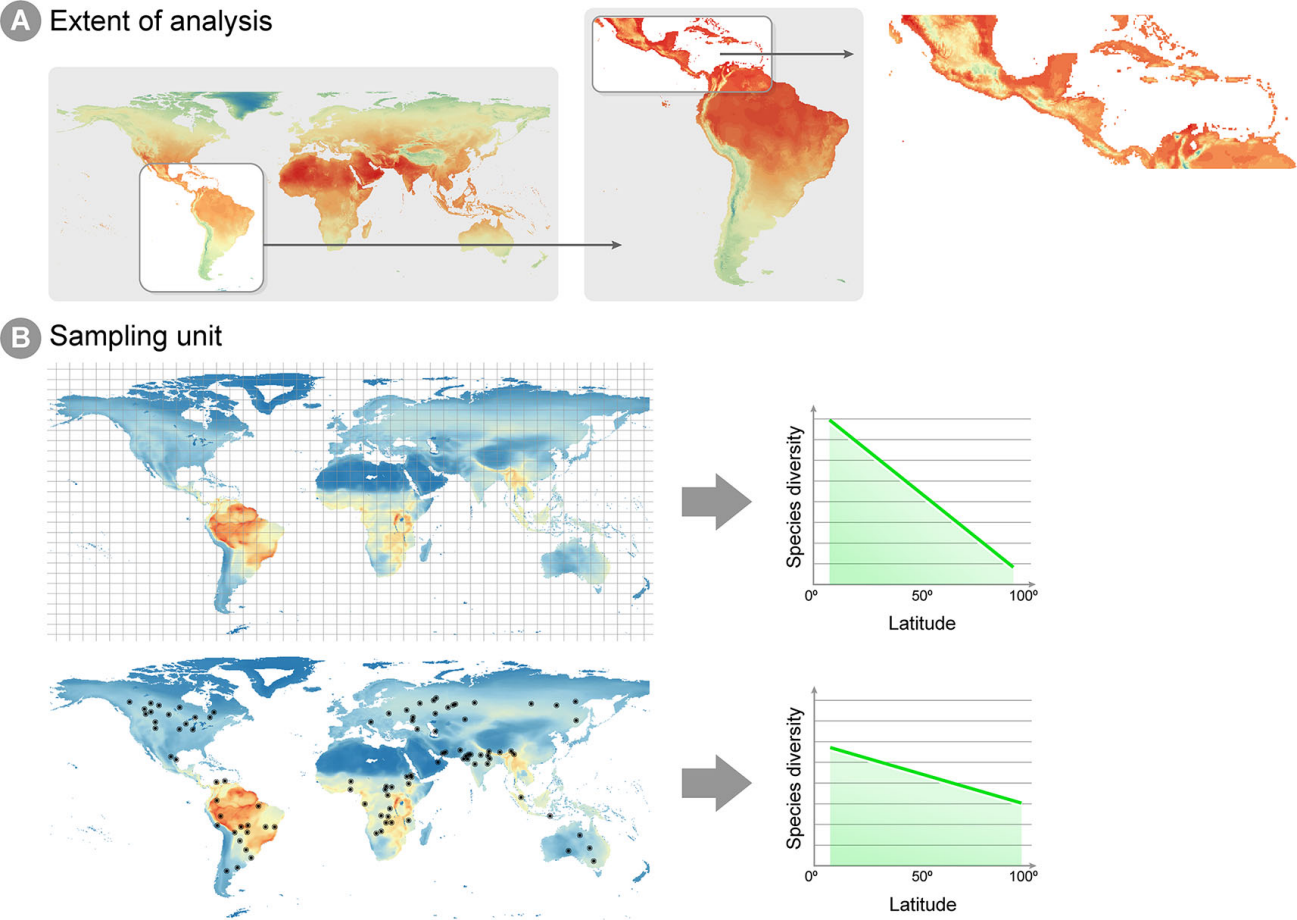
Spatial extent and sampling unit in macroecological analyses. **(A)** Different spatial extents can be analyzed in a macroecological study, which will reflect on the environmental information available for inference and how much extrapolation can be derived from the conclusions of the study. The figure shows annual mean temperature per cell, ranging from low temperatures in blue and high temperatures in red. Notice that the lowest temperatures (blue and green cells) are different for each extent. For instance, when studying Central America, the lowest temperatures can be found in Mexico highlands, whereas an extent focused on the whole Neotropics show lowest temperatures around the Andes mountains. Therefore, caution is necessary when inferences from studies on the Central America are extrapolated to the Neotropics extent. **(B)** Example of two different sampling units in macroecological studies: equally distant squared grids and local sites unevenly distributed through the globe. As highlighted by Hillebrand (2004), squared grids consist of a value averaged across sites within the grid, which decreases the effect of local scale factors (e.g., biotic interactions, dispersal and stochasticity) on the latitude gradient diversity pattern.

An issue arising in all studies addressing microbial macroecology is the proper evaluation of spatial and temporal scales under investigation. The idea that ecological patterns are scale-dependent is pervasive in ecological theory (e.g., Levin, 1992; Crawley and Harral, 2001; Chase and Leibold, 2002; Wu et al., 2002). Two macroecological studies (Willig et al., 2003; Rahbek, 2005) performed at different spatial scales reported distinct patterns for how species richness was associated with latitude and altitude. Hump-shaped patterns dominate species richness and altitude relationships, when the scale of the gradient survey is higher than 1,000 km, but is an uncommon pattern when the scale is below this value. The two studies cited above define two attributes of the sampling design that determine the scale that is being analyzed (**Figure 1**): the unit of sampling and the geographic space covered. The sampling unit is determined by the grain or focus size, i.e., the size of the common analytical unit in the analysis, whereas the geographic space covered, also called the extent, represents the geographical space on which inferences can be made (**Figure 1A**), in other words, the spatial extent covered by all sampling sites (Rahbek, 2005). Macroecological studies investigate processes in large geographical spaces, e.g., continental or global scales (Fierer and Jackson, 2006; Fuhrman et al., 2008; Nelson et al., 2016), which in general define a large extent for macroecological inference. The unit of sampling is represented by the degree of resolution in both response and predictor variables utilized, which can vary widely across studies. Communities' abundance or richness profiles (the response variable) might represent samples in a specific point in space, or samples across different spatial points in the same assumed community (**Figure 1B**). Equally, a single value in a predictor variable (e.g., abiotic conditions, such as temperature, pH, altitude, humidity, etc.) might represent either a 1 km^2^ or a 10 km^2^ geographic area, depending on how coarse the available environmental information is (Nottingham et al., 2018). The choice and evaluation of the available information is an important step in macroecological studies and may have a deep impact on the results obtained.

Several processes that might be important at local scales may have little effect on, and sometimes even confuse, a pattern at larger spatial scales. For example, Hillebrand (2004) compared studies on the latitudinal species richness gradient, a long well-recognized macroecological pattern, where species richness was known from occurrences equally sized squared areas equally distributed across space (i.e., grids) and studies where species richness was known from sampling points from different studies unevenly distributed across the globe (i.e., local sites). The results demonstrated that the decline of diversity towards higher latitudes was steeper in grid-based studies, suggesting the pattern is easier to detect by using a coarse-grained metric of diversity (as exemplified **Figure 1B**) because local processes (e.g., biotic interactions, dispersal and stochasticity) are averaged out. Additionally, microbial communities seem to be spatially structured mostly at larger study scales (Soininen, 2012), since such scales encompass multiple biogeographical regions separated by dispersal barriers and large variation in climate (Martiny et al., 2006). Therefore, at a smaller spatial scale, community composition may seem stochastic, or greatly vary in short periods of time. The overall conclusion from these studies is that different predictor variables will be biologically relevant at different ecological scales. This suggests that selection a set of predictor variables for model calibration must take into account the ecological scale of the investigated process. Traditionally, in macroecological species distribution models, temperature and precipitation have been successfully used as predictors for macro-organisms, although recent approaches have successfully incorporated biotic interactions into such models (e.g., Araújo and Luoto, 2007; Wisz et al., 2013). A remaining question is whether these same variables are biologically relevant for micro-organisms at large scales. At least for specific and microbiologically diverse ecosystems such as soils, climate—expressed both in terms of climatic factors such as temperature and precipitation, as well as climate-associated attributes such as soil pH, aridity and productivity—is considered a key driver of the structuring and functioning of global microbiomes (Delgado-Baquerizo et al., 2016; Delgado-Baquerizo et al., 2018; Bastida et al., 2019).

There are two main aspects of micro-organisms, which suggest that biologically relevant variables to predict micro-organisms' distribution may indeed be different from those used for macro-organisms. First, micro-organisms exhibit a higher evolutionary rate. Second, due to the organism size, the spatial scale at which micro-organisms perceive the environment is different from that of macro-organisms (Barberán et al., 2014). The first of these aspects indicates that micro-organisms readily adapt to new environments, which means that the distribution range of different microbial taxa is likely to be in equilibrium with environmental variables, which is not always true for macro-organisms (Araújo and Pearson, 2005). Additionally, a high evolutionary rate in micro-organisms indicates that temporal variability in microbiome composition may be high: when environmental changes occur, the microbiome structure is rapidly modified in response, whereas such responses in macro-organisms (expressed in the arrival and disappearance of species, as well as the rise of new adaptations in native species) may take a longer time. This suggests that each microbial sampling is invariably a narrow temporal snapshot of the microbiota, highlighting the importance of time-series sampling to describe for macroecological trends. The very reduced organism size implies that micro-organisms interact with different aspects of the environment, indicating that relevant predictor variables might include, but are certainly not restricted to, large-scale environmental variation. This is still a debatable topic in macroecology of micro-organisms, as some studies argue that micro-organisms respond to continental-scale climatic and environmental variation (e.g., Barberán et al., 2014; Delgado-Baquerizo et al., 2018), whereas others highlight that microscale environmental variation might be more important in predicting distribution patterns (Hendershot et al., 2017). Therefore, when implementing microbiome modeling, one should keep in mind that there is no consensus on which predictor variables should be used. For micro-organisms, the word “environment” might reflect both biotic and abiotic factors surrounding individuals of a species in a defined area, and the relative importance of these two types of factors might be different from what is known for macro-organisms.

The differences between micro- and macro-organisms need to be considered when implementing any of the methods described in this review. For each approach, it is necessary that the macroecological question is clearly stated, and in a way that the scale of sampling and the scale of the studied processes are in agreement with the scale of the proposed questions. In the following sections, we discuss different macroecological approaches for microbiomes, focusing on the description of macroecological patterns and the modeling of microbiomes at macroecological scales. In each case, we highlight how available methods and information can help researchers to answer questions at different spatial and temporal scales.

## Describing the Microbiome in Macroecological Scales

### Taxonomic Profiling and Exploratory Analyses in Microbial Macroecology

The basic input data for macroecological studies is a matrix displaying the presence-absence or abundance data of a biological entity in any taxonomic level across different sampling units (usually a locality defined by a pair of coordinates, but may reflect finer or coarser areas, depending on the specific question, Shade et al., 2018). For microbial communities, such a matrix is usually obtained through the taxonomic annotation of several short DNA sequences (i.e., *reads*) derived from the high-throughput sequencing of an environmental sample (Riesenfeld et al., 2004; Hugenholtz and Tyson, 2008). Reads must first be filtered according to quality and to remove possible contaminants, in order to minimize annotation errors; these tasks can be accomplished using tools such as Prinseq (Schmieder and Edwards, 2011) and Trimmomatic (Bolger et al., 2014). A common and desired practice is to deposit filtered reads in public repositories along with associated metadata, providing public access to the information. This is particularly important for macroecological studies, which make use of secondary data for analysis at large spatial scales. The most prominent repositories for metagenomic data are the NCBI short read archive (SRA; Leinonen et al., 2011b), MG-RAST (Meyer et al., 2008) and the European Nucleotide Archive (ENA; Leinonen et al., 2011a), some of which also provide bioinformatics tools for taxonomic annotation and statistical analysis (e.g., MG-RAST and MGnify; Mitchell et al., 2018). Is worth mentioning that the metadata standard for sequences deposited in International Nucleotide Sequence Database Collection (INSDC) is MIxS (Yilmaz et al., 2011).

**Figure 2:**
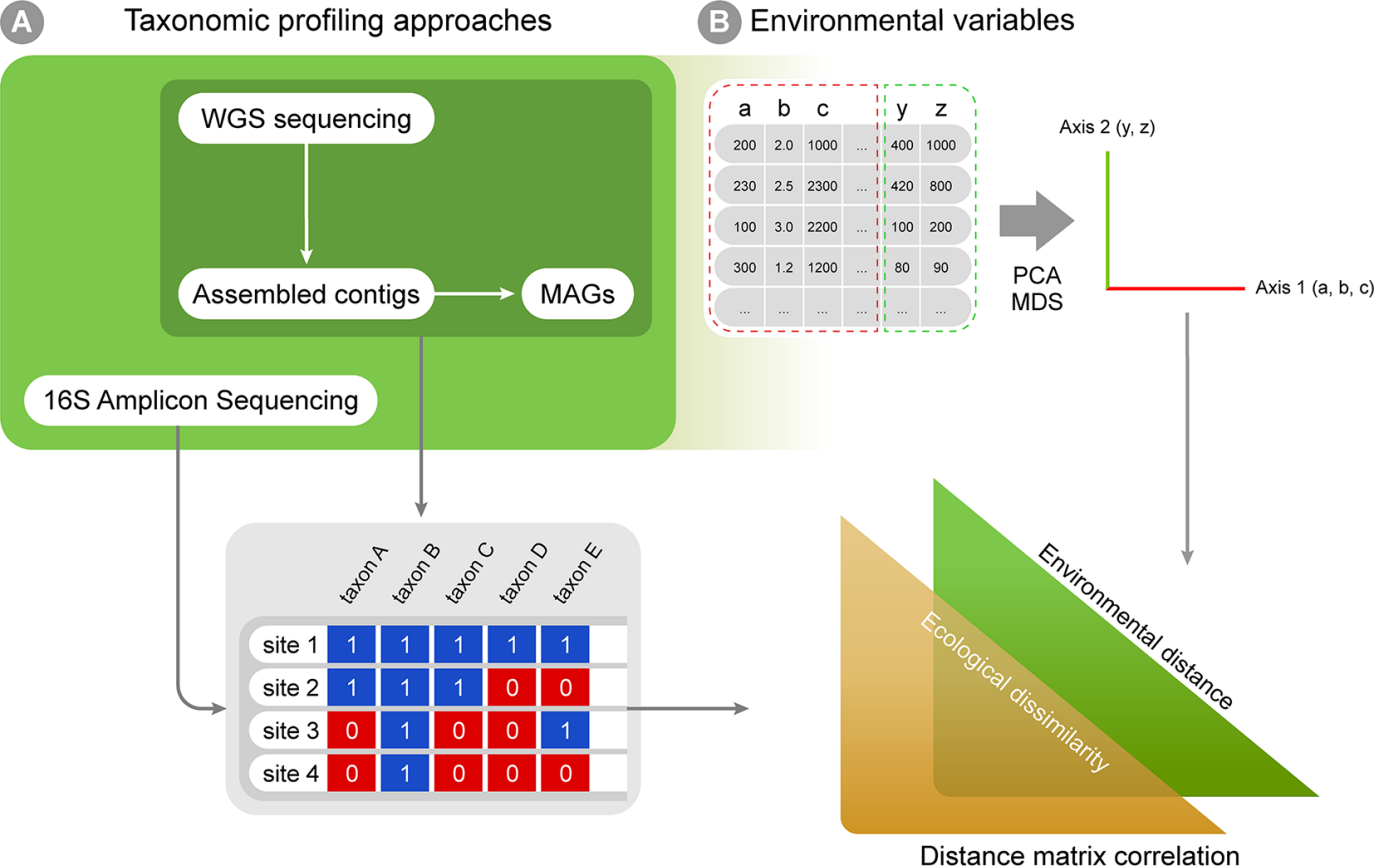
A workflow summary for taxonomic annotation and exploratory analyses. Taxonomic annotation methods are used to generate, for instance, presence-absence matrices **(A)**, which can be combined with environmental variables into correlation analyses **(B)**. The biological variation in environmental variables can be simplified through ordination analyses (such as PCA and MDS). Finally, distance matrices can be created for both ecological and environmental variation, and distance matrix correlation can be used to infer if environmental distances correlate with ecological differences among sampling sites.

Multiple approaches currently exist for obtaining taxonomic profiles from metagenomic sequences, and they mostly fall into four categories depending on the type of data used: 1) amplicon reads, 2) Whole Genome Shotgun (WGS) sequencing reads, 3) assembled contigs and 4) Metagenome-assembled Genomes (MAGs; **Figure 2A**). Each of these has unique advantages and limitations and is suitable to address different scientific questions (**Table 1**). Amplicon analysis consists mostly of PCR amplification of the 16S rRNA gene through the use of degenerate primers designed to cover as much of the diversity of Bacteria and Archaea as possible (Schmidt et al., 1991; McDonald et al., 2012). Next, amplicon sequences are mapped to reference databases, such as RDP (Cole et al., 2014), SILVA (Quast et al., 2013) and Greengenes (DeSantis et al., 2006), which contain pre-computed high-quality alignments of 16S rRNA genes, allowing for fast taxonomic assignments for millions of sequences. This approach tends to be accurate at low taxonomical levels (e.g., genera) and is cost effective, considering the coverage of sequencing per sample, making it possible to sample many more replicates per study. On the other hand calculating taxa abundances across samples can be a limitation due to the presence of multiple copies of the 16S rRNA gene in a single genome. Additionally, the so-called universal primers used for amplicon analysis usually do not amplify genes derived from major fractions of the diversity of Bacteria and Archaea, such as the candidate phyla radiation (Hug et al., 2016a).

**Table 1:** Approaches for obtaining taxonomic profiles from metagenomic samples.

Input type	Software	Speed	Reference Databases	Confidence	Advantages
Amplicon	Qiime, MOTHUR	Fast	SILVA, RDP and Greengenes	Low	Extensive databases of sequences and samples for comparison
WGS Homology	Diamond, BLAST, BLAT, MEGAN	Slow	nr, Uniprot, pfam	Medium	Based on the whole genetic diversity
WGS K-mer	Kraken, FOCUS	Fast	RefSeq Genomes	Medium	Based on the whole genetic diversity
Assembled Contigs	Assembly: SPAdes, ID-BA_ud, Ray-Meta Contig Classification: CAT, MEGAN, Kaiju	Slow	nr, Uniprot, pfam	High	Discovery of new taxa, more reads assigned
MAG	Assembly: SPAdes, IDBA_ud, Ray-Meta Binning: Metabat, GroopM, ABAWACA, CheckM Classification: CAT/BAT	Slow	N/A	High	Yields draft or complete genomes, discovery of new taxa, more reads assigned

One common alternative to amplicon sequencing is Whole Genome Shotgun (WGS), i.e., the sequencing of DNA fragments covering the whole diversity of genes in an environmental sample. Similar to amplicon based studies, WGS reads are annotated by comparing them to previously characterized sequences deposited in reference databases, encompassing genes from multiple taxa. This comparison can be based on homology or the search for similar k-mer profiles (i.e., the set of all possible sub-strings of different lengths for a DNA sequence). Due to redundancy in the genetic code, proteins are more conserved than nucleotide sequences; using homology to detect similar protein sequences is more sensitive and suitable for detecting distant evolutionary relationships, allowing more sequences to be classified. Because the degree of identity between the sequences of naturally occurring microbes and those available in reference databases is often very low, annotations of WGS reads often require using permissive cutoffs (i.e., reads are assigned to a taxon even if the identity is low, e.g., only 30%), provided that it falls within other assumed cutoffs of alignment, length and e-value. Several reference databases are currently available, as well as tools to detect protein-protein and protein-nucleotide homology (**Table 1**). As an alternative to homology searches, k-mer composition profiles are significantly faster and make it possible to rapidly analyze a large number of samples (**Table 1**).

Using WGS sequencing further allows for the assembly of raw reads into larger contigs, and, in some cases, later binning into metagenome-assembled genomes (MAGs; **Figure 2**). This approach may improve taxonomic classification by assessing longer genomic fragments that derive from such sequence assembly. The Critical Assessment of Metagenome Interpretation (CAMI) challenge reviewed several metagenomics tools (Sczyrba et al., 2017). This study distinguished between taxonomic binners (which allow taxonomic abundances to be inferred by clustering individual sequences, then assessing longer genomic fragments Lin and Liao, 2016; Wu et al., 2016), from taxonomic profilers (which focus on predicting a taxonomic abundance profile without necessarily classifying every sequence, often assessing only raw reads Ounit et al., 2015; Koslicki and Falush, 2016). They show that classifiers in general were more accurate than profilers in estimating the relative abundances of taxa. This increased performance is due to the fact that longer sequences contain more phylogenetic information than short reads, leading to less noise in the taxonomic profile. Moreover, because sequence assembly reduces the total volume of sequence data to be classified, more sensitive homology searches that are computationally more demanding may be applied than the rapid searches that are used for classification of short, raw reads. Two recently developed tools that explicitly exploit the added information in assembled contigs are MEGAN-LR (Huson et al., 2018) and the Contig Annotation Tool [CAT, (von Meijenfeldt et al., 2019); https://github.com/dutilh/cat] that exploit all sequences in the full GenBank reference database for taxonomic classification. A limitation of metagenomic assembly is that it is susceptible to possible errors arising during the assembly, which is aggravated when population diversity of the sampled microbial community is high (Sczyrba et al., 2017). Moreover, high levels of sequence heterogeneity between related strains may lead to abundant genomes in the sample being misassembled as chimeras, and potentially misclassified. The subsampling of shotgun metagenomic reads before assembly has been applied to resolve this problem (Hug et al., 2016b).

Once contigs have been assembled into longer fragments of the genomes present in the community, metagenome-assembled genomes (MAGs) may be reconstructed by binning contigs from the same genome together. Several software tools are available to perform MAG reconstruction (**Table 1**). At this stage, phylogenetic and phylogenomic methods can be used to determine the taxonomic affiliation of these MAGs with even more confidence than that of individual contigs. Additionally, MAGs and assembled contigs can be used to build custom sample-specific reference databases for read mapping (e.g., Speth et al., 2016). The main advantage of using such databases is that often many more reads can be assigned, because the contig sequences represent the strains that are reconstructed from the same sample, minimizing the occurrence of false positives. Therefore, the obtained taxonomic profile contains less noise and more comprehensively represents the data.

The taxonomic profiles obtained from the methods above can be assembled into presence-absence or abundance matrices and further explored using classic multivariate exploratory analyses, such as multivariate ordination/canonical methods (Hanson et al., 2012; Xue et al., 2018). Under the macroecological rationale, exploratory analyses are used to describe the biological variation across a global or continental gradient in potential explanatory variables (e.g., describing diversity or abundance variation across the latitudinal temperature gradient, continental atmospheric variation, etc.; Shade et al., 2018). Correlation among explanatory variables is a common issue in biological statistics, and multivariate ordination is then used to reduce dimensionality and yield new mathematically uncorrelated axis from the original correlated explanatory variables (Legendre and Legendre, 2012; **Figure 2B**). A few approaches widely used for this purpose are: 1) Principal components analysis (PCA), which is based on covariance or correlation matrices and is suitable for sets of linearly correlated measures; 2) principal coordinates analysis (PCoA), which differs from the PCA by extracting eigenvalues from similarity or distance matrices, therefore being appropriate for non-linear relationships; 3) multidimensional scaling (MDS) that, unlike PCA and PCoA, is not based on eigenvalues decomposition and, like PCoA, is limited to Euclidean distances matrices and 4) correspondence analysis (CA), based on contingency table of categorical variables (Bray and Curtis, 1957; Clarke, 1993). The new mathematical axes provide a mathematical space where measurements from the actual environmental samples can be placed and compared. The associations between variables (e.g., diversity and temperature) can also be tested by classic statistical analyses like regression and correlation, which can be based on both original explanatory variables and new mathematical axes created by ordination analyses. Additionally, ecological similarity between localities can be explored using distance measures (e.g. Euclidean, Mahalanobis, Jaccard, and Bray-Curtis) and compared against a distance matrix for a potential explanatory variable in the same localities and statistical significance can then be assessed by using a test such as the Mantel test (**Figure 2B**). Such approaches are commonly used in macroecological studies to statistically assess the correlation between two distance matrices based on variables of interest (e.g., Duarte et al., 2009; Bell, 2010).

### Describing Community Structure With Co-Occurrence Networks

**Figure 3:**
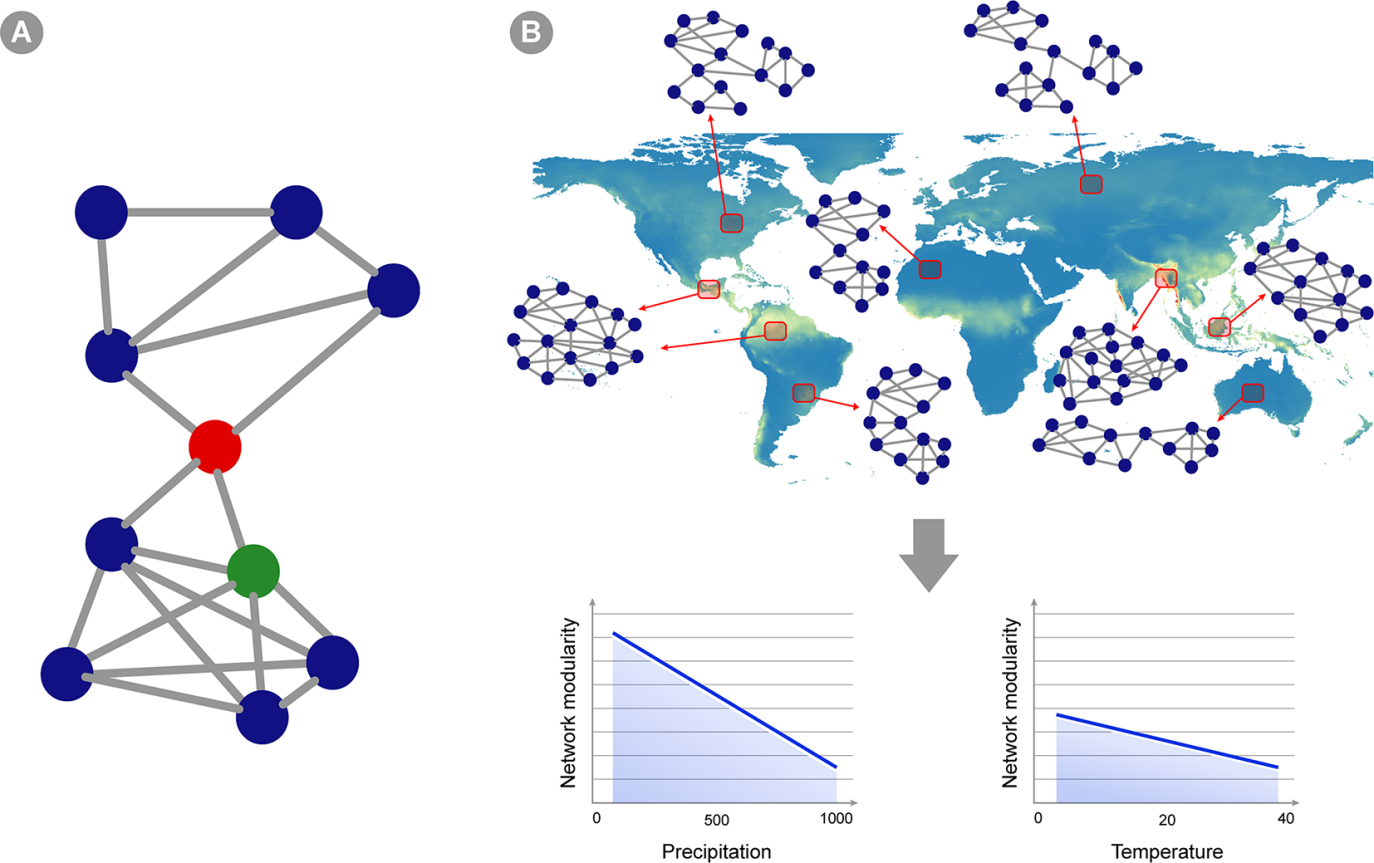
Co-occurrence networks applied to microbial macroecology. **(A)** A hypothetical example of a co-occurrence network. Circles represent different taxa and edges connecting two circles indicate statistically significant co-occurrence between those two taxa, i.e., they co-occur more than expected by chance in the set of samples analyzed. Network structure can indicate ecosystem properties, and these can be translated into statistics summarizing network topology (see Box 1). For instance, this hypothetical network shows two subunits (or modules) separated by the taxon indicated as a red circle. This taxon is also a node with high betweenness centrality (i.e., indirect connections between any two nodes in the network has a high probability of going through this node), whereas the green circle represents a node with high degree (i.e., showing a connection to many other taxa). **(B)** A hypothetical example of a macroecological study using co-occurrence networks. Red squares represent an area where several samples were gathered and analyzed, yielding a single abundance matrix and a corresponding co-occurrence network (two sites pointing to the same network represent areas in which networks are highly similar). The topology of the network changes in different ecosystems across the globe, and the overall hypothetical pattern is represented in the graphics below: network modularity (i.e., defined as the number of subunits within the network, as well as the relative proportion between connections within and between modules) decreases as precipitation and temperature increases (but the change is less intense for temperature).

Co-occurrence networks (CNs) has been used to describe associations within microbial community (**Figure 3**). Usually, in these networks, the nodes represent taxa and the edges represent statistically significant positive or negative correlations in the abundance of taxa across several samples in a given environment or host (Faust and Raes, 2012). A few authors may also include abiotic factors as nodes (e.g. Li et al., 2017). Using CNs can reveal insights about possible ecological interactions and distribution patterns of microbial taxa (Faust and Raes, 2012; Cardona et al., 2016). Two important types of information can be retrieved from CNs: 1) changes in community structure across environmental gradients, that is, variation not only in the species abundance, but especially in the degree of correlation between taxa across environmental gradients; and 2) potential biotic interactions that can be useful for macroecological modeling (*Predicting Microbial Distribution and Community |Composition*). Since CNs are based on abundance correlation, it is desirable that they are built over a large number of sampling units, and therefore hold great potential for application in macroecological studies (Berry and Widder, 2014). Distinct approaches have been used to construct CNs and derive information from their structure, such as distance or similarity matrix metrics among the samples used to construct the networks (Fan et al., 2018; Jackson et al., 2018; Marasco et al., 2018; **Box 1**). Overall, the same matrix generated by the software tools listed in the previous section can be used as input for CN calculation. Samples can be grouped according to the macroecological variable of interest (e.g., temperature variation across latitudes, atmospheric variation across a continent, variation in land cover across the globe) and the structure of CNs from each of these groupings can be compared across global or continental scales (**Figure 3**). Note that comparison of microbial community structure has often been performed across different ecosystems (e.g., comparing the structure of networks between fresh and saline water environment), but the macroecological approach supports the rationale of a comparison within the same environment (e.g., soil samples) across an environmental gradient (e.g., temperature, pH, etc.; Barberán et al., 2012). Several measures exist to describe network structure, such as symmetry, degree distribution, checkerboard index (Horner-Devine et al., 2007; Araújo et al., 2011; Layeghifard et al., 2017), but the best usage of such metrics is an ongoing debate (Layeghifard et al., 2017) and is highly dependent on the ecological question being asked.

Box 1 **Building and Interpreting Co-Occurrence Networks.**

Several tools are available to build and interpret co-occurrence networks. The software CoNet (Faust and Raes, 2016), developed in Cytoscape (Shannon et al., 2003), allows the usage of several measures for dependency, similarity and dissimilarity, to build and visualize co-occurrence networks. In order to build these CNs, the microbial composition data is provided in relative abundances. Some annotation tools provide microbial composition in read counts, in this case one can use SparCC (Friedman and Alm, 2012), which calculates abundance correlations among taxa without the issues associated with compositional data (Mendes et al., 2018), for further CNs analysis. Alternatives to SparCC are REBACCA (Ban et al., 2015) and CCLasso (Fang et al., 2015). Kurtz et al. (2015) presented another tool: SPIEC-EASY, a pipeline that transforms relative abundance data and estimates interaction graphs. Finally, a few approaches are based on information theory, for instance: using mutual information combined with other metrics, implemented in CoNet (Lima-Mendez et al., 2015). Choosing a correlation method for network construction is critical once networks generated by different methods can provide contrasting results (Weiss et al., 2016). Methods should be chosen taking into consideration if microbial community data are presented as relative abundance or in absolute read counts.

*Keystones in CN*

There is no consensus on the operational definition of keystone for microbial ecology (reviewed in Banerjee et al., 2018). However, a usually proposed definition is that keystones are highly connected microbial taxa presenting a unique and crucial role for community structure and functioning, so their loss or removal should have large impacts on microbial community (Banerjee et al., 2018). In this sense, network theory provides us with quantitative ways to characterize how connected a given microbial taxa is. One criterion, based in network theory, to determine a putative keystone taxon is high betweenness centrality (BC; e.g., Lupatini et al., 2014; Banerjee et al., 2016; Jiao et al., 2016; Li et al., 2017; Mendes et al., 2018), albeit an investigation based on dynamical modeling found lower BC to be correlated with higher probability of a taxon being keystone (Berry and Widder, 2014). The BC of a node A is the number of shortest paths connecting two nodes which pass through the node A. Nodes with high BC connect portions of the network that would otherwise be sparsely or not connected at all. Therefore, removing high BC nodes leads to a sparser network, disconnecting modules in several cases. The number of connections a node presents, which is called the node's degree, is also a frequent metric used as a keystone index (Comte et al., 2016; Hartman et al., 2018). This is based on the idea that, taxa (nodes) that are connected with multiple others are important to network structure, and their potential removal would have a high impact to the community. It is interesting to highlight that, whereas one node can have both high degree and high BC (in which case this taxa would be considered keystone by both definitions), it is also possible to find nodes in which BC is high and degree is low, or vice-versa, leading to a disagreement between these two keystones definitions. Therefore, it is important to have in mind the biological process of interest because this will determine the more important features in a given community and what keystone definition one should use.

A different approach, based on metabolic networks (Guimera and Amaral, 2005), assumes that the network is formed by modules (i.e., semi-independent groups of cohesive, interacting taxa). In this approach, one can calculate the z-score, which is a measure of the number of interactions a taxon has within its module; and the c-score, which describes how evenly distributed are the interactions of a given taxon across multiple modules. These two values allow us to classify the taxa in network hubs (z-score > 2.5; c-score > 0.6), module hubs (z-score > 2.5; c-score < 0.6), connectors (z-score < 2.5; c-score > 0.6) and peripherals (z-score < 2.5; c-score < 0.6) (Poudel et al., 2016; Fan et al., 2018). Putative keystones taxa would then be the nodes identified as network hubs, module hubs and connectors. One advantage is that this definition takes into account multiple features that might make a node important to a network (e.g., participating in a network within a hub or as connectors between hubs), whereas, when one looks only at BC or high degree, a single type of keystone feature is taken into account.

*Indirect Effects From CNs*

In networks, species that do not directly interact can influence each other through cascading effects that spread through the network (indirect effects). Guimarães et al. (2017) developed an analytical framework to quantify the total amount and the importance of the indirect effects in a given network. Their results show that network structure is what drives how the indirect effects spread through the network (Guimarães et al., 2017). Networks of micro-organisms, which are species-rich networks formed by a small core of highly connected species and many species poorly connected (Banerjee et al., 2018), are predicted to show a higher amount of indirect effects than poor, highly modular networks. Therefore, quantifying indirect effects might be an important aspect in the study of which micro-organisms are keystones to a given community relevant to maintain relevant ecosystems functions and contribution to resilience and stability in face of global environmental changes (Berry and Widder, 2014).

In addition to measuring indirect effects, it is possible to explore the consequences of such effects. Resilience and stability are important aspects of network structure that can be measured by using approaches derived from the study of dynamical systems. Coyte et al. (2015) proposed an extremely general and suitable framework that can be used to analyze species-rich microbial networks. Their approach uses the eigenvalues of the matrix that describes the effects of ecological interactions at the equilibrium (Jacobian matrix) associated to a given network, to analyze the stability and resilience of microbiome networks. Their approach can be used in networks that possess any combination of different types of interactions (cooperation, competition, exploitation, amensalism and commensalism). One important result of their analyses is that cooperation tends to destabilize microbial networks. The destabilization effect happens because of the presence of positive feedbacks between the species when they cooperate, which leads to cascading effects. For example, a decrease in population size of one species might lead to all the species they positively interact with to decrease as well. On the other hand, competition gives a stabilizing effect in the network; compensating the destabilizing effect that increasing richness can have in an ecological community (May, 1972).

Co-occurrence networks may also be used to identify keystone taxa (**Box 1**). The keystone concept was first coined by Paine (1966), who demonstrated that the removal of the sea-star *Pisaster ochraceus* caused a dramatic change in community structure on a rocky shore, concluding that the species functioned as an important element for maintaining community integrity, most likely due to its non-redundant role (Paine, 1969)⁠. This definition can be applied in the microbial ecosystem and be empirically investigated by using network approaches. Keystone taxa can be compared across macroecological scales to investigate whether and how the importance of specific groups as key taxa in communities across an environment varies on global scales. Since keystone taxa usually perform important and non-redundant functions, their identification may be important to understanding ecosystem functioning.Thus, an approach coupling keystone identification with measurements of functional diversity across macroecological scales holds potential to bring numerous insights (see below). Finally, another insight derived from CNs is how the network structure may favor or constrain cascading effects (**Box 1**), which may favor or imperil the resilience of the communities against perturbations (another ongoing debate within ecosystem ecology; Oliver et al., 2015). Cascading effects often propagate across networks, connecting the dynamics of taxa that do not directly interact with each other. In fact, networks of taxa are subject to influences from taxa they directly interact with, as well as to indirect effects that pervade the network, i.e. from taxa with which they do not interact directly. Under certain conditions, indirect effects can be more important to the network dynamics than the direct effects (Ohgushi, 2005). Indirect effects can be measured across macroecological scales to assess, in a spatially explicit manner, in which ecosystems indirect effects seem to play a more important role to maintain microbial community stability (Guimarães et al., 2017).

### Revealing Macroecological Patterns From Microbiome Functional Diversity

Functional ecology, defined as the study of the roles that organisms play in their ecosystems, also holds great potential for microbial macroecology. Studies investigating levels of functional diversity across macroecological scales are already common for macro-organisms (Fu et al., 2017; Jarzyna and Jetz, 2018)⁠, both in theoretical investigations of processes determining functional diversity (Safi et al., 2011) and in more practical inquiries such as the conservation of ecosystem functions (Devictor et al., 2010). Yet similar studies have not been performed for micro-organisms. For instance, previous studies have explored like global patterns of mammalian functional diversity (Safi et al., 2011) as well as global scale marine macroecological patterns (Amend et al., 2013) have no equivalent investigation concerning microbial functional diversity. Macroecological studies might yield insights on the patterns observed for the functional diversity of micro-organisms across different environments in the globe, and address their relation to ecosystem functioning and service provision (Mace et al., 2012).

Functional diversity is one of the three main biodiversity dimensions investigated in macroecology, alongside taxonomic and phylogenetic diversity (Webb et al., 2002; Devictor et al., 2010). Functional diversity is usually defined as the amount, variation and distribution of traits in a community (Dıaz and Cabido, 2001), originally measured by the calculation of the total branch length of the functional dendrogram constructed from information about taxa' functional traits (Petchey and Gaston, 2002). From this initial method, several new conceptual and mathematical approaches have been developed and implemented (a few revised in Petchey et al., 2004), but none of them dismiss the need to 1) choose the functional traits through which organisms will be distinguished, 2) define how the diversity of the trait information will be summarized into a measure of functional diversity, and 3) validate the measurements through quantitative analyses and experimental tests (Petchey and Gaston, 2006). In micro-organisms, functional traits are usually viewed as the genetic and biochemical characteristics of organisms affecting ecosystem functioning, such as the production of metabolic inhibitors or enhancers, or enzymes playing a role in ecosystem metabolic pathways (Dıaz and Cabido, 2001). In this sense, the function of micro-organisms in an ecosystem is defined by their genetic composition, which ultimately dictates the molecules they metabolize (Faure and Joly, 2016). Similar to taxonomic annotation, functional traits can be derived by direct functional annotation of metagenomic short-reads from an environmental sample (with no taxonomic annotation). Alternatively, prior metataxonomic approaches (e.g., 16S rRNA) can be used to taxonomically assign individuals in a sample, and then functional annotation can be derived from their phylogenetic position. Software tools to perform both approaches are summarized in **Table 2**, with their respective references. All of these metagenomic and metataxonomic functional annotation approaches are based on genomic databases and the accuracy of annotation depends on the quality of software databases. Furthermore, many genes are still unassigned, and their functions are unknown, making it challenging to infer ecological functions from genetic content alone (Faure and Joly, 2016).

**Table 2:** Tools used to annotate functional potential profiles from metagenomic reads or to infer them from 16S taxonomic annotation.

Tool	Approach	Synopsis	Features	Reference
BLASTx	Read annotation	Uses alignment approach to annotate nucleotide reads into potential proteins	+ great sensitivity - it can be very slow for high-throughput data	Altschul et al. (1990)
MetaGeneAnnotator	Read annotation	Identify putative proteins by estimating di-codon frequencies through the GC content of a nucleotide read	- not precisely estimate de Domain of a given sequence	Noguchi et al. (2006)
DIAMOND	Read annotation	Uses double indexing alignment to annotate nucleotide reads into potential proteins	+ 2000 to 20000 times faster than BLASTx	Buchfink et al. (2015)
SUPER-FOCUS	Read annotation	Functional profiling of metagenomes	+ output consists in a three hierarchical level functional profile, useful to choose your level of functional resolution	Silva et al. (2016b)
MGS-Fast	Read annotation	Preprocess and analyses WGS reads into functional profiles by using stringent DNA-DNA matching to the IGC database.	+ includes preprocessing steps (read trimming and removal of low-quality sequences) and taxonomic profiling	Brown et al. (2019)
MetaCLADE	Read annotation	Uses a multi-source domain annotation strategy to profile reads into protein domains.	+ designed to also annotate metatranscriptomic reads	Ugarte et al. (2018)
PICRUSt	16S inference	Uses evolutionary modelling to predict community putative functional profiles from 16S marker gene using a genome reference database	+ online interface to users unfamiliar with programming	Langille et al. (2013)
PAPRICA	16S inference	Places reads into a 16S phylogenetic tree of consensus genomes to predict the functional profile	+ very accurate to infer functional profile of well-known organisms that have plenty of genomes in the database	Bowman and Ducklow (2015)
FAPROTAX	16S inference	Extrapolates community taxonomy into putative functional profiles	- database used from cultivated organisms only	Louca et al. (2016)
QIIME	Functional pipeline	Provides a wide range of microbial assembly analysis and visualizations from raw nucleotide sequences	+ network and phylogenetic analysis and core assessment	Caporaso et al. (2010)
MOCAT2	Functional pipeline	Assemble and quality-filter reads to comprehensively predict them functionally and quantify them	+ also annotate metagenomes taxonomically	Kultima et al. (2016)

The degree of functional diversity has been used to investigate two main macroecological patterns in microbial communities: 1) relationships between community taxonomic and functional composition among microbial communities (Louca et al., 2016; Vieira-Silva et al., 2016; Galand et al., 2018) and; 2) how microbial functions vary in time and space (Dinsdale et al., 2008; Ren et al., 2017; Galand et al., 2018). Usually the most accessed functional measures are diversity (including functional richness, evenness and divergence), composition, redundancy and rarity. Several algorithms and computational tools have been published in order to assess and quantify these functional features (**Table 3**, also reviewed in Mouchet et al., 2010; Schleuter et al., 2010; Song et al., 2014; Bond-Lamberty et al., 2016; Ricotta et al., 2016). Addressing the above-cited questions, one of the emerging patterns in micro-organisms is a decoupling between functional and taxonomic composition (Louca et al., 2016). This trait suggests that microbial communities may present a high degree of functional redundancy, meaning that shifts in taxonomic community composition do not lead to shifts in functional community composition. It has been hypothesized that the mechanisms underlying microbial assemblage are distinct from mechanisms governing functional composition, and that environmental factors are potential predictors of functional composition (Louca et al., 2018). We further suggest that approaches for characterizing functional diversity should also be coupled with estimates of function turn-over and nestedness; metrics that in macroecology are commonly used to measure shifts in species composition mostly along abiotic gradients, the so called *beta-*diversity (Legendre et al., 2005; Anderson et al., 2006; Jost, 2007). This information would allow us to answer questions such as whether a specific subset of functions is filtered and maintained in a specific environment; or how functions are changing across abiotic gradients.

**Table 3:** Tools to calculate functional diversity features.

Tool	Approach	Synopsis	Features	Reference
PHYLOCOM	Software	Calculates trait distribution to compare with random community consortia as well as uses evolutionary models to simulate trait and phylogenetic evolution	+ uses null models to test hypothesis of trait similarity + integrates trait information with evolutionary analysis + able to deal with polytomies	Webb et al. (2008)
FDiversity	Software	Focuses on calculation of functional diversity indexes and statistically analyze them	+ user friendly interface + accepts different input data formats	Casanoves et al. (2011)
FD	R-language package	Uses functional dispersion index and measures diversity based on distances of traits in a multidimensional space	+ allows missing values on calculation + allows weighting traits per abundance	Laliberté and Legendre (2010)
SYNCSA	R-language package	Uses matrix correlation to estimate trait patterns, phylogenetic signal and environmental variations for metacommunities	+ allows environmental characteristics to be considered	Debastiani and Pillar (2012)
cati	R-language package	Estimates community assembly patterns by species interactions and environmental filtering	+ allows differentiation among individuals + can integrate phylogenetic information into analysis	Taudiere and Violle (2016)
funrar	R-language package	Estimates functional rarity based on abundance and/or spatial frequency of species	+ estimates functional uniqueness, distinctiveness and taxon scarcity and restrictedness	Grenié et al. (2017)

## Predicting Microbial Distribution and Community Composition

**Figure 4:**
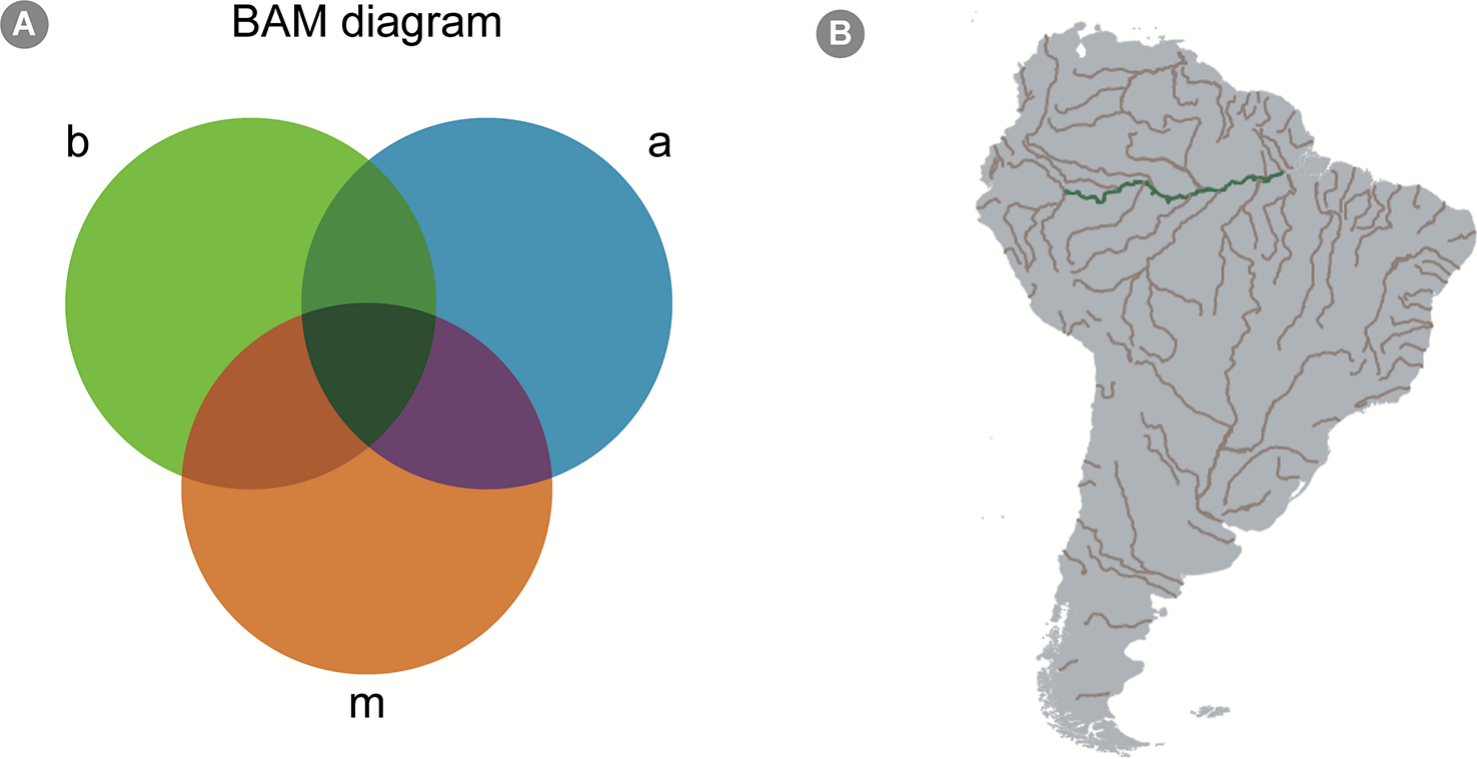
The BAM Diagram. **(A)** A scheme of a hypothetical BAM diagram (abbreviation for “*biotic, abiotic, and movements*”), highlighting the intersection between the different aspects determining the presence-absence of species. The *b* circle, colored in green, represents biological aspects allowing the presence of the species; the *a* circle, colored in blue, represents the abiotic aspects; finally, the *m* circle, colored in orange, represents the movement aspect, which consists in the dispersal capacity of the species. The intersection represents areas where more than one of those aspects allows the existence of the species. For instance, the green intersection represents an area where both biotic and abiotic conditions allow the species to exist, but the species is unlikely to disperse to that area. Similarly, the purple intersection represents an area where abiotic conditions allow the species to exist and is within the species' dispersal capacity; however, biotic conditions (for example, presence or absence of important species with which it interacts) do not allow their existence. All species occur only in areas represented by the dark green intersection, i.e. the intersection of all three factors. Mathematical models, however, can calibrate species niche based, solely on abiotic factors (which is the case of most SDM approaches), and, in these cases, the BAM diagram is a good conceptual framework to interpret the results. **(B)** A geographical projection of the BAM diagram for a hypothetical microorganism in South America. The grey areas across the continent represent sites to where the species can potentially disperse to (based on the idea that micro-organisms have high dispersal capacity, see *Predicting Microbial Distribution and Community Composition* in text). Assuming our hypothetical species prefer freshwater conditions, rivers in South America are colored in brown, to represent the intersection between factors *a* and *m* in the diagram. Finally, the green color of the Amazon river indicates an area where all factors allow the existence of the species (i.e., the species can disperse to the area, it is a freshwater environment, and it shows biotic conditions favorable to its establishment, e.g. the presence of specific species with which it cooperates).

Macroecologists describe spatial patterns of biodiversity aiming to ultimately create accurate models that can predict biodiversity under different scenarios. The patterns described are analyzed, and the underlying biotic and abiotic drivers of species distribution and abundance are tested in a statistical framework. Understanding the mechanisms behind these patterns allows macroecologists to predict biodiversity in geographic areas not yet studied, contributing to decrease biodiversity shortfalls (Hortal et al., 2015) as well as how biodiversity would respond to changes in the environment (Kerr et al., 2007). The BAM (as an abbreviation for ‘*biotic, abiotic and movements*') diagram is a conceptual framework used in macroecological modeling to summarize the determinants of species distribution on global scales (**Figure 4**; Soberón and Nakamura, 2009).

In the BAM framework, the presence of a focal species in a specific site is determined by: (1) the presence, absence and/or abundance of other species in the same environment (i.e., biotic factors, the B in BAM); (2) the availability of the environmental attributes that are suitable for the focal species (i.e., abiotic factors, the A in BAM) and; (3) the focal species capacity to migrate into biotically and abiotically suitable areas (i.e., movement capacity, the M in BAM; **Figure 4**). This idea is described in a more formal manner in the Hutchinsonian concept of ecological niche, i.e., the n-dimensional hypervolume in which a species can exist (Colwell and Rangel, 2009; Holt, 2009; **Figure 4**). This conceptual framework is important for models that attempt to predict the occurrence of taxa, since it highlights which factors are expected to affect taxa presence in different locations. For macroorganisms, models are usually calibrated with the usage of abiotic factors at large spatial scales, specifically temperature and precipitation, which were shown to be good predictors of terrestrial species distribution range (e.g., Soberón, 2010). Such models usually show acceptable accuracy, but several studies highlight the importance of accounting for migration capacity and species interactions in distribution modeling (Araújo and Luoto, 2007; Wisz et al., 2013).

When it comes to micro-organisms, it is necessary to clearly understand which factors affect the distribution of microbial species. The BAM diagram offers an adequate conceptual framework to start addressing this question. Several authors have suggested that the dispersal capacity of micro-organisms is much higher than that of macroorganisms (Finlay and Clarke, 1999; Martiny et al., 2006; Barberán et al., 2014). In this aspect, the movement feature of the BAM diagram would have little effect on the distribution of species, since several studies indicate that micro-organisms are highly dispersive (Bovallius et al., 1980; Fenchel and Finlay, 2004; Martiny et al., 2006; Barberán et al., 2014; but see, e.g., Peay et al., 2010), and that spatial structuring of microbial communities are only perceivable on large spatial scales. This leaves us with the biotic and abiotic factors as major drivers of micro-organisms' distribution. As previously discussed in *Conceptual Challenges for Transitioning Across Spatial and Temporal Scales*, a few studies have highlighted the importance of different abiotic factors in structuring microbial community, which are not always related to the environmental predictors used in distribution modeling of macroorganisms. Such variables include, besides temperature and precipitation, edaphic conditions, soil pH and concentrations of different chemical molecules (Lauber et al., 2009; Drenovsky et al., 2010; Zhou et al., 2016). Additionally, the biotic interactions among species have been advocated as important determinants of species occurrence (Larsen et al., 2012; Ramirez et al., 2015; Ramirez et al., 2018). Therefore, in the following sections we describe how to access available spatial-explicit environmental data for micro-organisms modeling, as well as modeling approaches that can account for both biotic and abiotic factors.

### Using Abiotic Variables to Model Microbial Communities

Each sample taken from the environment is under the influence of a huge number of variables in many spatial and temporal scales. In order to model the composition of microbiomes, and therefore the distribution of micro-organisms across the globe, it is important to have available environmental data on the relevant spatial and temporal scales. The variables used to model micro-organisms will depend on the specific environment under study. Micro-organisms living in the soil are affected by different environmental factors than those living in a freshwater lake or in the ocean. This is different than what is seen for macroorganisms, where global temperature and precipitation play major roles defining biogeographic realms (McGill, 2010). While acknowledging that global variation in temperature and precipitation might define biogeographic areas for micro-organisms (Martiny et al., 2006), we argue that this definition will differ when comparing between micro-organisms living in different environment types (e.g., soil vs freshwater micro-organisms).

Physical properties are usually important in several environments, such as temperature, precipitation, moisture and solar radiation. These variables can be measured or modeled *via* remote sensing platforms and remote sensing-based modeling tools. Due to the advent of environmental monitoring satellites and the creation of on-line data processing and distribution platforms, there is a wealth of environmental data with global coverage available to the general public, ranging from raw satellite images to validated measurements of parameters, such as land surface temperature, precipitation rates, the concentration of gases such as CO_2_ in the troposphere and photosynthetic activity (**Table 4**). These databases contain climatic spatially explicit information such as land surface temperature, net primary productivity, vegetation and leaf area indexes, evapotranspiration, detailed landcover map and precipitation rate. Additionally, since other aspects of soil and atmosphere might also be necessary to fully characterize the abiotic environment of micro-organisms. Information pertaining to soil physical (e.g., clay content) and chemical (e.g., pH) conditions, as well as soil classification across the globe can be retrieved from these databases. Similarly, when investigating the atmosphere microbiome, the atmospheric chemical composition may play a large role on community composition by changing the chemical properties such as pH and playing an important role on ecological processes, such as nitrification (Keller et al., 2006; Hutchins et al., 2009; Hatzenpichler, 2012). An example of atmospheric chemical composition data available, such as the products based on the Atmospheric Infrared Sounder (AIRS), is a hyperspectral instrument on board of Aqua satellite (**Table 4**). By decomposing the infrared radiation in 2,378 bands, AIRS can provide daily measurements of trace components abundances in the atmosphere, including ozone, carbon monoxide, carbon dioxide, methane, and sulfur dioxide in different strata of the atmosphere, among other parameters (Morgan et al., 2004; Maddy et al., 2008; Xiong et al., 2008; Engelen et al., 2009; Lin et al., 2013).

**Table 4:** Databases for spatially explicit abiotic ecological data for use in community modeling.

Database	Data	Synopsis	References	Data access
Atmospheric Infra-Red Sounder (AIRS)	Greenhouse gases concentration in troposphere (CO2, CO, CH4, O3); etc.	Provides atmospheric chemical composition measurements by decomposing the infrared radiation in 2378 bands	AIRS Science team and Texeira, 2008; Morgan et al., 2004; Maddy et al., 2008; Xiong et al., 2008; Engelen et al., 2009; Lin et al., 2013	https://search.earthdata.nasa.gov
Tropical Rainfall Measuring Mission (TRMM)	Precipitation	Precipitation rate and rainfall rate. Was operational from 1997-12-01 to 2015-03-31	Wilheit et al., 1991	https://search.earthdata.nasa.gov
GPM (Global Precipitation Measurement)	Precipitation	Global observations of rain and snow. Operational from 2014-03-01 until the present	Hong et al., 2004; Huffman et al., 2007; Stocker et al., 2018	https://search.earthdata.nasa.gov
MODIS (Moderate Resolution Imaging Spectroradiometer)	Land surface temperature; Vegetation idexes (NDVI, EVI, LAI); Primary production; Evapotransiration; Ocean chlorophyll; etc…	Produces a huge list of high precision environmental products, with high temporal resolution, that are validated with field data	Cohen et al., 2003; Didan, 2015; Friedl and Sulla-Menashe, 2015; Giglio et al., 2015; Running et al., 2017; Savtchenko et al., 2004; Turner et al., 2006; Wan et al., 2015	https://search.earthdata.nasa.gov
SOILGRID	Bulk density; Soil granulometry; Soil classification; Cation exchange capacity; Soil organic content; pH; etc…	Models a set of soil's physical and chemical properties through the combination of soil samples data with a large set of soil covariates using machine learning techniques	Hengl et al., 2017	https://soilgrids.org
GLDAS—Global Land Data Assimilation System Version 2	Rain precipitation rate; Evapotranspiration; Root zone soil moisture; Soil moisture (in various depths); Soil temperature(in various depths); etc.	Models land surface states and fluxes using optimal fields. Includes 40 climatic parameters with temporal coverage from 1979-01-01 to present with high temporal resolution	Rodell et al., 2004; Rodell et al., 2009; Kumar et al., 2006; Peters-Lidard et al., 2007	https://search.earthdata.nasa.gov
WorldClim Version2	Annual Mean Temperature; Mean Diurnal Range; Temperature Seasonality; Temperature Annual Range; Annual Precipitation; Precipitation Seasonality; etc…	Set of 19 bioclimatic variables averaging of climatic parameters from 1970 to 2000, modeled through general circulation models (GCM).	Fick and Hijmans, 2017	http://worldclim.org/version2
WorldClim 1.4 downscaled (CMIP5) data	The same as WorldClim Version2 projected to the future	Future projections for the same WorldClim 19 bioclimatic variables for two periods, 2050 (average for 2041–2060) and 2070 (average for 2061–2080), based Intergovernmental Panel on Climate Change (IPCC)	Stocker, 2014	https://www.worldclim.org/cmip5v1

Furthermore, the data gathered from satellites and ground observations, are used in the parameterization of climatic models, which allows the calculation of additional climatic variables. The Global Land Data Assimilation System (GLDAS) is a good example of this kind of climatic modeling (Rodell et al., 2004; Rodell et al., 2009). It models land surface states and fluxes, using advanced land surface modeling techniques based on optimal fields (Rodell et al., 2004). Currently GLDAS includes datasets from four land surface models implemented in NASA's software LIS (Land Information System), namely Mosaic, Noah, the Community Land Model (CLM), and the Variable Infiltration Capacity (VIC), resulting in massive archive maps of up to 40 climatic parameters, water and energy flux, as well as underground temperature and moisture, with maximum depth of 1.1 m and with temporal coverage ranging from 1979-01-01 to nowadays (Kumar et al., 2006; Peters-Lidard et al., 2007). Another good example of a climatic model available is the Worldclim, one of the most used climatic datasets in ecological modeling. It comprises a set of 19 climatic variables relevant to many ecological processes, with a global coverage of 1000 m spatial resolution (Fick and Hijmans, 2017). This set of variables is a result of the averaging of climatic parameters from 1970 to 2000, modeled through the usage of general circulation models (GCM), which are suitable to model worldwide geographic variation in ecological processes that respond to spatial patterns of climatic heterogeneity. The calculation methods to produce this set of variables were implemented in R and are available through the function *biovars*, from the Package ‘dismo', version 1.1-4 (Hijmans et al., 2017). In addition, Worldclim also provides future projections for the same set of 19 climatic variables for two periods, 2050 (average for 2041–2060) and 2070 (average for 2061–2080), based on the set of models used in the Fifth Assessment Report of the Intergovernmental Panel on Climate Change (IPCC) for the four scenarios of greenhouse gases concentration (Stocker et al., 2014). These future projections provided by Worldclim have the advantage of being bias corrected, using the current climate Worldclim data as base line, making the three sets of variables compatible. In addition, the AIRS, TRMM, GPM, and GLDAS products are available in NASA’s Goddard Earth Sciences Data and Information Services Center (GES DISC), which is part of the Earthdata platform, specialized in processing and distribution of climatic data.

Given the huge amount of climatic and environmental data available to the global landscape, microbial ecologists are now using those same analytical tools used in traditional macroecological studies. This allows them to select the most important drivers in predicting microbial diversity distribution patterns and to predict the structure of microbial communities across the globe, thereby accessing cause and effect associations. In these efforts, machine learning approaches, especially classification or regression Random Forest analysis and structural equation modeling (SEM) should be highlighted (Breiman, 2001; Grace, 2006). Specifically, Random Forest analysis constitutes specific algorithms of statistical methods of classification and regression trees (CARTs) that use binary division or regression, respectively, to form a set of trees where the importance of each predictor is inferred by decreased prediction accuracy through the random permutation of the values of these predictors (Liaw and Wiener, 2002; Wei et al., 2010). SEM routines are then used in microbial ecology studies coupled with Random Forest in order to reveal the relation between those ‘*a priori*' selected abiotic drivers and the target-variable in question, such as the Shannon Index, used as a proxy for microbial diversity (Delgado-Baquerizo et al., 2016). Therefore, SEM is a valuable alternative when the objective is to detail the specific relationships between multiple predictors and the modeled variable, separating them as individual pathways in the network of relationships that characterizes natural systems (Delgado-Baquerizo et al., 2017).

### Incorporating Biotic Interactions in Modeling Microbial Communities

Another important issue in macroecological modeling is the inclusion of biotic interactions as predictor variables. There is an increasing evidence that species interactions improve the explanatory and predictive power of species distribution models, based on environmental variables for macroorganisms (Araújo and Luoto, 2007). Usually the inclusion of biotic interactions in species distribution models is based on previous biological knowledge of the studied species and uses a limited number of species/taxa per model, while considering their geographical distribution (Araújo and Luoto, 2007; Wisz et al., 2013; de Araújo et al., 2014). These models are usually based on species distribution models and use a maximal entropy approach—e.g., Maxent for modeling (Phillips and Dudík, 2008). However, there are also integrative modeling approaches that incorporate co-occurrence patterns into species distribution models (Pollock et al., 2014). Other modeling techniques use machine learning approaches, such as neural networks, which do not make assumptions related to species occurrence probabilities and linear relationships among environmental and biological variables, and so provide more realistic assemblage models (Harris, 2015).

Studies with micro-organisms have also suggested that including biotic interactions is necessary to build suitable predictive models (Larsen et al., 2012). However, despite their importance, these interactions can be elusive to detect, and unraveling the interactions network in microbial communities is an ongoing challenge (Faust and Raes, 2012). Biotic interactions can be inferred to some extent from co-occurrence networks (*Describing Community Structure With Co-ccurrence Networks*), but the increase of computational capacity and the development of accurate machine learning and network modeling methods has made possible to explore new approaches to statistically assess biotic interactions from large abundance datasets, such as Bayesian networks (BNs) and Genetic Algorithms (GA). The BNs are graphical models consisting of a set of variables (represented as nodes in the network) and directed arcs that describe the sets of conditional dependencies between these variables, as well as the joint probability distribution among then (Pearl, 2014; see also **Figure in Box 2**). The variables set in BNs may be both abiotic factors as well as biotic interactions, and the model can be calibrated with the same input abundance matrices generated by taxonomic annotation methods (*Taxonomic Profiling and Exploratory Analyses in Microbial Macroecology*). Additional columns representing abiotic aspects of each sampling site can be added to the abundance matrix to represent the abiotic environment experienced by a specific microorganism. This approach allows the creation of species distribution models by taking into account both biotic and abiotic aspects simultaneously in a model across large geographical scales (Staniczenko et al., 2017). These models can be further used to predict the change in the abundance of an organism when any other node (either an abiotic aspect or another species abundance) changes in the environment. A few microbial studies have already used a BN approach to study, e.g., the bacterial diversity in gut microbiota for patients with psoriatic arthritis (Scher et al., 2014) and the gut microbiota in HIV positive patients (Vázquez-Castellanos et al., 2015). Similarly, in macroecology, a few studies have used the BN approach, e.g., for range prediction of California grassland community (Staniczenko et al., 2017) and assessment of threat status of pacific walrus population in Russian and Alaskan waters at four different time periods (scenarios) throughout the twenty-first century (Jay et al., 2011).

Box 2 **Bayesian Networks: Advantages and Drawbacks.**

Bayesian networks show several advantages that support their recent application in complex fields, such as: 1) network modularity, being able to integrate multiple ecosystem components (Chen and Pollino, 2012; Nojavan et al., 2014; Nojavan et al., 2017; Uusitalo, 2007), such as in management decisions field, where it is possible to integrate several sub-models as social, ecological and economic aspects (Chen and Pollino, 2012); 2) the capability of dealing with complex and nonlinear systems (Uusitalo, 2007; Aguilera et al., 2011; Phan et al., 2016; Beuzen et al., 2018); 3) possibility of incorporating expert knowledge (Uusitalo, 2007; Aguilera et al., 2011; Alameddine et al., 2011; Death et al., 2015; Phan et al., 2016), through blacklists (i.e., unrealistic relationships that are not allowed in the model) and whitelist (i.e., relationships already known in the literature); 4) being able to use a small number of samples (Uusitalo, 2007; Phan et al., 2016) 5) simplicity and little difficulty in interpreting outputs, even for non-modelers (Aguilera et al., 2011; Death et al., 2015); 6) being a rather “open” approach, different from other methods, which can be considered complicated “black-box” approaches (Chen and Pollino, 2012); 7) being able to handle high dimensional systems with the proper number of samples (Aguilera et al., 2011); 8) dealing with missing data through conditional probabilities or Bayes theorem (Uusitalo, 2007; Aguilera et al., 2011; Death et al., 2015), and finally 9) presenting less computational cost to analyze and compare different scenarios, such as climatic changes, by setting variables states in the model (Chen and Pollino, 2012; Death et al., 2015).

The main weakness of the BN approach is the lack of feedback possibilities in the model, due to it being directed acyclic graph (DAG; Phan et al., 2016). This can be bypassed by integrating models. The most critical drawback pointed in most studies is the discretization of continuous variables (Uusitalo, 2007; Aguilera et al., 2011; Nojavan A. et al., 2014; Death et al., 2015; Phan et al., 2016). The principal argument is that it causes an inevitable loss of information from data, linear relationships and consequently model performance (Uusitalo, 2007; Nojavan A. et al., 2017; Beuzen et al., 2018). However, using discrete values allows for better modeling of non-linear relationships between variables, as well as complex distributions such as bi- or multimodal distributions and can introduce greater robustness against error (Hartemink, 2001). As alternatives, there are models that could handle continuous data and not have mathematical restrictions, such as Mixture of Truncated Exponentials (MTE) models and the BN created for continuous variables (Qian and Miltner, 2015). However, it is hard to find simple examples and they are not easily found in any commercial software, which makes implementation difficult for non-modelers.

**Figure In Box 2:**
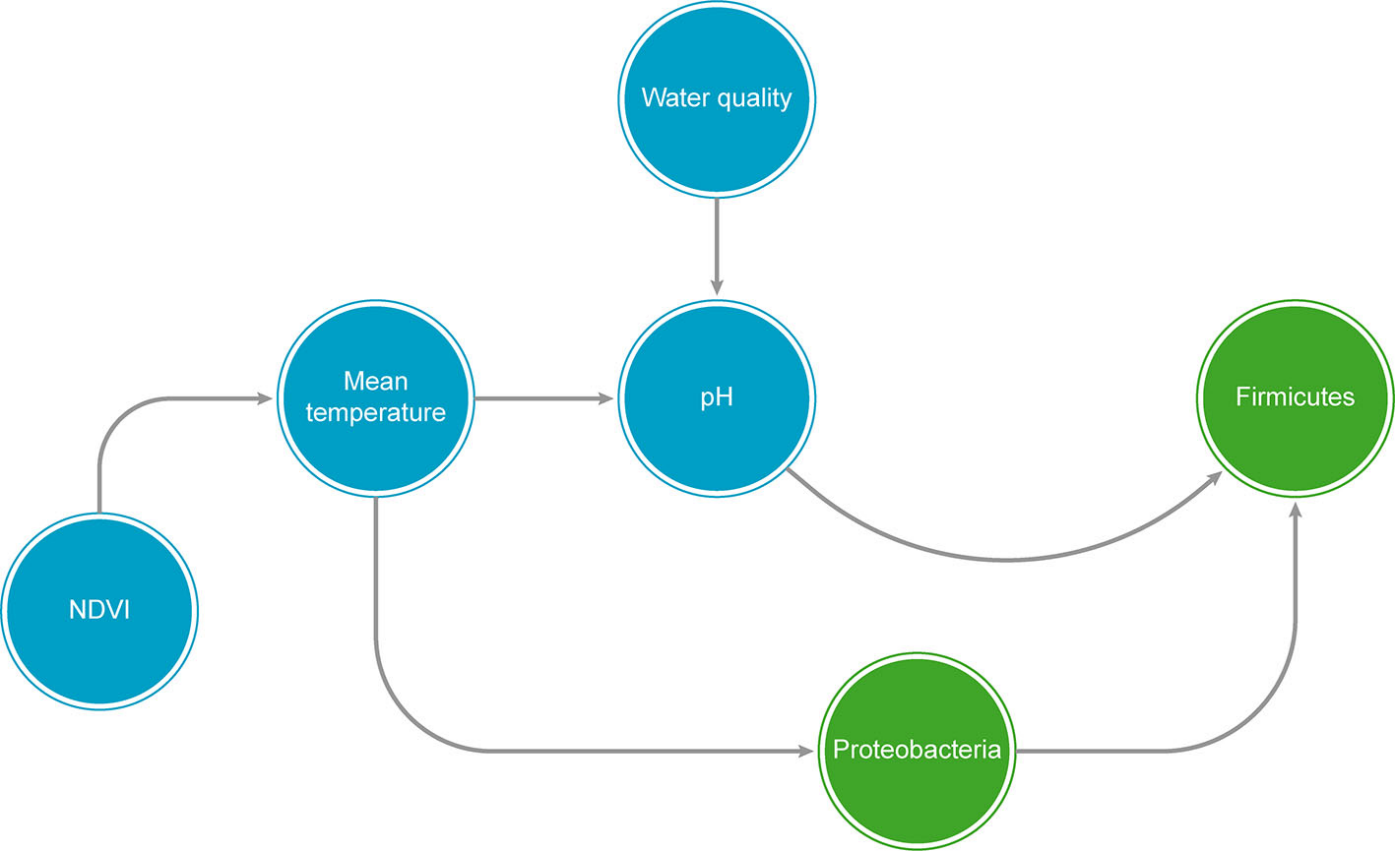
A graphical example of a hypothetical Bayesian Network (BN), showing both biological taxa (green circles) and predictor abiotic variables (blue circles). NDVI = Normalized difference vegetation index.

Similarly, the use of predictive models based on the genetic algorithm (GA) method holds great potential to infer microbial interactions but has not been explored by microbiologists so far, to the best of our knowledge. The GA is an approach to solve problems inspired by the process of natural selection. Genetic programming (GP) is a particular type of GA that can be used to generate computational artifacts, such as computer programs, mathematical models, and logical models, that help to explain an observed data (Koza, 1992). The GP approach usually starts from a population of programs (algorithms) that show random levels of success in solving a task (in this case, describing the significant biotic interactions observed in a microbiome dataset). The fittest programs, that is, those best describing the data, are selected for reproduction and may undergo some “mutation” according to predefined parameters. This process is repeated over several generations in an analogy to natural selection, and the final generations are expected to show a population of much fitter programs than the initial ones. This procedure is essentially a heuristic search technique that looks for an optimal or at least suitable program among the space of all programs available. Since the construction of the models is totally guided by data, without the need of *a priori* hypotheses, the greatest potential of this technique is to generate hypotheses about the relationship between micro-organisms, as well as between micro-organisms and environment, that can be assessed by other approaches (such as BNs, dynamical modeling or common correlative statistics, described above). Applications of GP include designing electrical circuits (Koza et al., 2000), reverse engineering biochemical reactions (Sugimoto et al., 2005) and describing epidemiological relationships (Veiga et al., 2018).

Another promising approach to resolve microbial interactions is the use of dynamical models (Widder et al., 2016), which can bridge the gap between fundamental ecological knowledge and empirical interactions between taxa, by relying on explicit and mechanistically sound hypotheses. For such purpose, several modelling approaches are available (reviewed by Song et al., 2014 and by Succurro and Ebenhöh, 2018), each presenting its own set of assumptions concerning biotic and abiotic components of community. The most widespread approach is assuming direct biotic interactions among taxa and representing these interactions by using the generalized Lotka-Volterra model (gLV). This is a particular case of the population dynamic model, which can then serve to investigate concepts related to community dynamics such as co-occurrence networks and keystone taxa (Berry and Widder, 2014; see **Box 1**). Some authors also advocate the use of metabolic-explicit dynamical models that integrate aspects of community and environmental variables, such as stoichiometry-based models and flux balance analysis (FBA; Song et al., 2014). While these approaches avoid black-box modeling and provide valuable insights into community functioning across environments, they present parameterization challenges, in gLV for instance, the number of parameters increases with the square of the number of interacting species, hindering model analysis. Future developments integrating dynamical modeling and statistical parameterization techniques are thus poised to improve the suitability of dynamical modeling approaches to exploration of microbial community interactions; meanwhile, dynamical modeling is readily available to investigate important subsystems with fewer interacting organisms.

### Species Distribution Modeling for Community Prediction

The steps described in *Using Abiotic Variables to Model Microbial Communities* and *Incorporating Biotic Interactions in Modeling Microbial Communities* allow us to highlight important abiotic environmental factors as well as biotic interactions necessary to model our focal microbial communities. Although few of the techniques presented, such as BNs, can model community composition on their own, another approach largely used in macroecology for this purpose is the set of modeling tools known as species distribution modeling (SDM). The use of SDM has been regarded as a well-established approach that can be used to overcome the lack of species spatial data, and holds great advantages for micro-organisms, a group in which the Wallacean deficit (i.e., the lack of information about species distribution) tends to be high. The SDM techniques are generally based on the concept of species ecological niches, which is the set of biotic and abiotic conditions that allows a species to persist indefinitely in a location (Soberón, 2007). Evidence so far suggests that biotic interactions should have a larger importance at smaller scales (but see Gotelli et al., 2010 and Araújo and Rozenfeld, 2013), while abiotic conditions, such as climate, should have a larger influence at larger spatial scales (McGill, 2010). Based on this, macroecologists have used the set of climatic conditions where a macroorganisms lives to estimate its potential geographic distribution. Whereas this is largely efficient for macroorganisms, more empirical evidence is necessary to evaluate these premises for micro-organisms.

**Figure 5:**
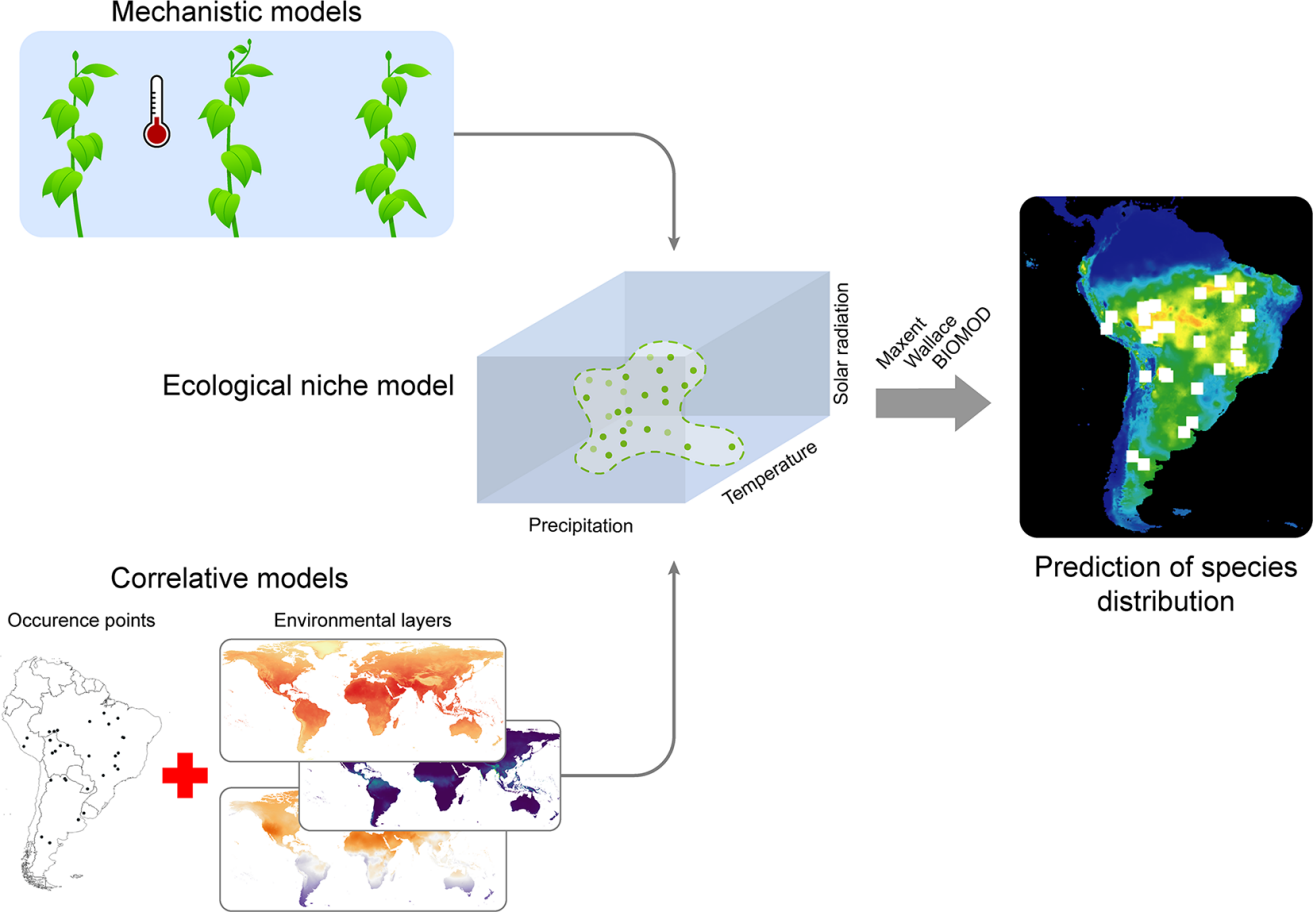
A workflow on techniques for species distribution modelling. Ecological niches can be modeled both by using mechanistic models (upper left figure, representing temperature laboratory manipulative experiments on plants) or by using correlative models (lower left figure, representing the use of spatial-explicit environmental data combined with the knowledge about occurrence points for the species). The ecological niche is then calibrated on an n-hyperdimensional volume defined by all predictor variables used in the study (only three dimensions are shown in the cube to the center). Green points indicate known occurrence for the species projected into the environmental space; dashed green lines represent the ecological niche inferred from those points. The inferred ecological niche can then be projected into geographical space, which consists on the geographical areas having environmental conditions within those inferred to be the species' niche (are highlighted as suitable areas for the species in the map). Since the niche is statistically calibrated, i.e., as a statistical relation between predictor environmental variables and presence-absence response variables, the final map shows a gradient of environmental suitability for the species across the space.

Two sets of approaches can be used for SDMs: the mechanistic and correlative species distribution modeling (**Figure 5**). Mechanistic SDMs use information obtained from *ex-situ* experiments that indicate the environmental conditions that a species can tolerate (e.g., maximum and minimum temperature). This information on physiological tolerances can then be used to map areas that are environmentally suitable for the species, which can be transformed into presence/absence information (Kearney and Porter, 2009; **Figure 5**). The lack of experimental information indicating species tolerance have limited the use of mechanistic approaches; however, in areas where experimental data is abundant, such as agricultural science, mechanistic models have been used to predict potential areas for determined crop varieties (e.g., Nabout et al., 2012). This approach can be potentially useful for microbial macroecology, since these organisms can be easily manipulated *ex-situ*, because of their small, short life span and large population sizes (Jessup et al., 2004).

The correlative approach, on the other hand, uses statistical associations between acknowledged species occurrences and environmental conditions to estimate the Grinellian Niche (**Figure 5**). The type of statistical model used for this approach is then chosen upon the type of occurrence data available: continuous (abundance data), binary (i.e., presence/absence data) or presence-only data (usually the latter, since abundance information is not always available and real absence data is challenging to confirm). Presence-only models of species distributions are largely used for macroecological studies, with several algorithms available, from simple ones, such as the BIOCLIM, up to more complex models based on machine learning techniques - e.g, Random Forest and MAXENT (Elith and Leathwick, 2009). While some authors claim that some algorithms have a better performance than others, the current view is that the choice of the algorithm also depends on the context in which SDMs are applied (see Peterson et al., 2010). Despite the known importance of abiotic conditions to determine large-scale species distributions, one must consider also current and historical movement limitations, such as geographical barriers, dispersal capacity and biogeographical history (Barve et al., 2011). However, it is still necessary to identify whether and how movement limitations are important to model microbial distributions, because of their overall high dispersal capacity.

Several computational tools can be used to apply SDMs, many of them freely available, open source, and collaborative (e.g., Naimi and Araújo, 2016; Kass et al., 2018). Microbiology can benefit from these methods in many research lines, since SDMs have been used not only to predict individual species distribution, but also species richness and composition (e.g., Guisan and Rahbek, 2011), species potential invasive areas (e.g., Smolik et al., 2010), as well as to understand niche evolution and speciation patterns (e.g., Silva et al., 2014; Silva et al., 2016a), and past species dynamics (e.g., Nogués-Bravo, 2009); and to model geographical range responses to climate change (e.g., Pecl et al., 2017). Specifically, SDMs present an important method to understand how species geographic range may respond to climate change. However, because of high microbial adaptation capacity, it may be a methodological challenge for microbiologists to incorporate evolution when trying to model species distribution into other time periods (Ofori et al., 2017).

## Conclusion

**Figure 6:**
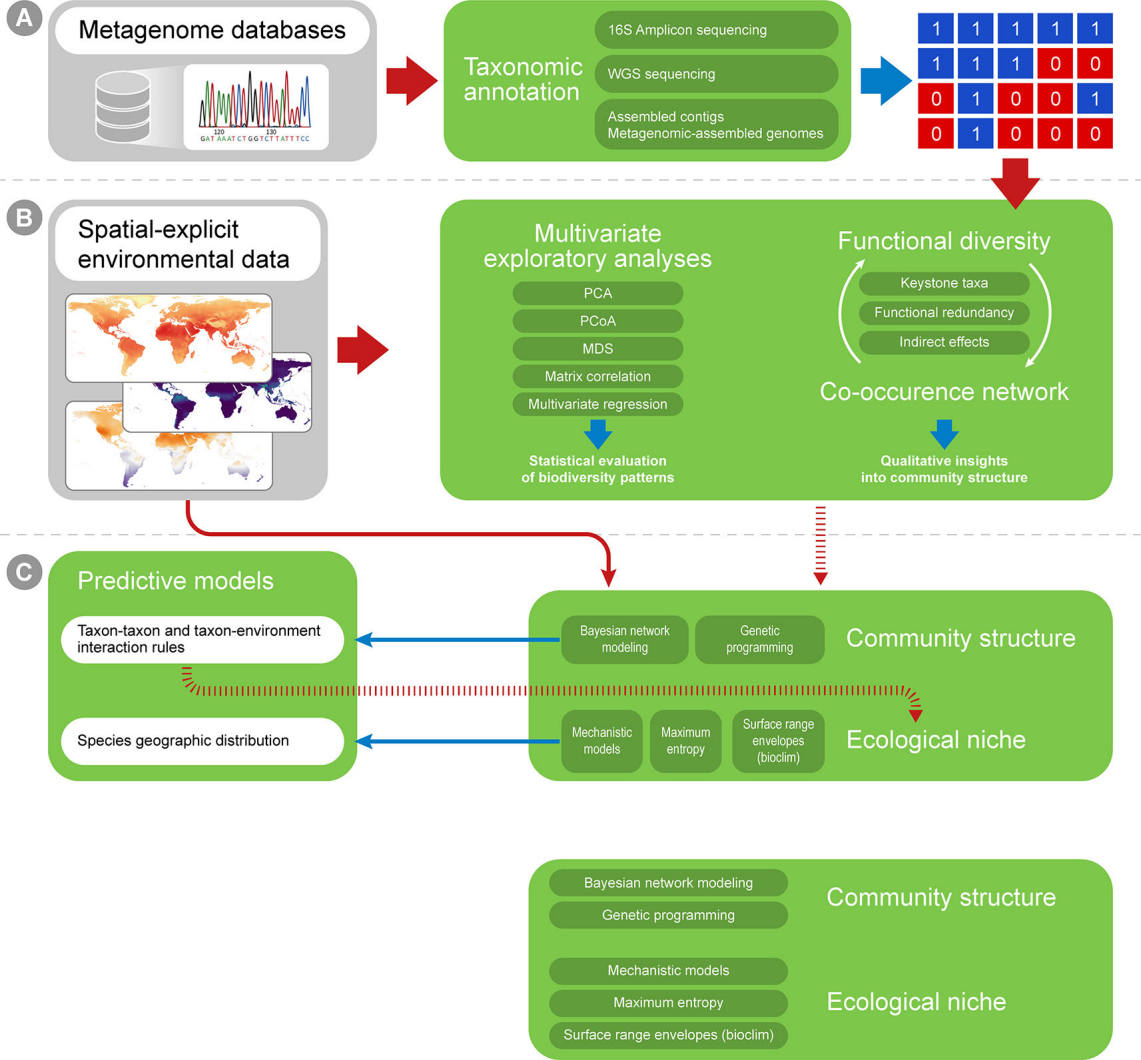
A methodological framework to investigate the macroecology of micro-organisms. The framework shows methods related to **(A)** gathering taxonomic data on environmental samples, **(B)** exploring the data with exploratory analyses as well as statistical tests (e.g., correlation and regression analyses), and **(C)** using the data to create predictive models about the presence/absence of species across different environments. Solid red arrows indicates input and output data that is used as input for analyses, and blue arrows indicate the output of these analyses. Dashed red arrows indicate data that can yield indirect insights for an analysis (although they are not commonly used as direct data input for the method). Grey boxes indicate external information sources and green boxes indicate the methodological approaches reviewed in this manuscript. Dark green boxes within green boxes indicate the specific techniques used in each approach. White boxes indicate the final outputs for the macroecological approach, i.e., models explaining how environment and biotic interactions affect species presence-absence and ultimately community composition. **(A)** Data from metagenomic databases can be annotated taxonomically to yield presence-absence or abundance matrixes for several ecosystems. **(B)** Spatial-explicit environmental data can be incorporated into exploratory analyses (such as PCA and MDS) as well as correlation analyses (such as regression and Mantel test) to investigate micro-organisms diversity patterns on global scales. Functional diversity can also be investigated on macroecological scales (both directly inferred from sequence reads or from the taxonomic annotation of samples). Co-occurrence networks are commonly used in microbiology studies and can yield interesting insights when different groups of samples are compared across an environmental gradient. The understanding of functional diversity and functional redundancy can be coupled with co-occurrence networks to infer the existence of keystone taxa, as well as the extent of direct and indirect effects throughout a network, and then describe the community structure and ecosystem functioning. Such structure can then be compared across macroecological scales (e.g., analyzing how the importance of specific taxa as keystone taxa varies across different environments). **(C)** Spatial-explicit environmental data can also be incorporated into models to understand community structure (such as Bayesian network modeling and genetic programming) as well as models to calibrate ecological niche (such as mechanistic and correlative niche models). These models can incorporate insights from analyses shown in **(B)**. Similarly, insights on biotic interactions, derived from community structure models, can be incorporated into ecological niche models (which commonly only use abiotic environmental variables as predictors). The final predictive models will allow microbiologists to understand interaction rules structuring microbial communities, predict the present of important taxa in different environments and infer microbial community composition across the globe.

The vast amount of microbial community data available represents an exciting prospect for advancing the field of microbial macroecology. In this review, we outlined the main questions in macroecology, community ecology and addressed how microbial ecologists can address them with bioinformatics, statistical and modeling tools. We covered fundamental aspects of biodiversity, reviewed classical approaches used in microbial ecology in a macroecological context, and highlighted the existing caveats and solutions to implement ecological modeling of microbial communities, which is a crucial research area for both the theoretical and practical aspects of macroecology. These approaches can serve as a general framework for microbial macroecology, addressing the two-part focus of macroecology: describing community patterns (and their drivers) at large scales and predicting community composition across the globe (**Figure 6**). The framework we present here consists of 1) gathering biological data to generate an abundance matrix, and environmental data to generate an environmental matrix; 2) exploring the associations between biological and environmental data at macroecological scales, using exploratory and network approaches; 3) incorporating insights from the previous step into modeling tools for community prediction.

The main difficulties for this research avenue are the theoretical implications derived from the biology of micro-organisms, such as higher dispersal capacity, higher evolutionary rate and the putative environmental drivers of community composition. New studies are necessary to address which environmental factors are relevant for modeling microbial distribution and to define whether the high dispersal capacity of micro-organisms makes this aspect uninformative for biogeographic patterns (i.e. the classic statement of “Everything is everywhere”). Also to evaluate whether the adaptive potential of micro-organisms is indeed high enough to violate the usual assumption of niche conservatism applied to ecological modeling. The insights from these future studies will have great impact on microbial ecological model interpretation. We predict that the development of modeling methods and approaches used in microbial macroecology, an exciting and flourishing field, will significantly contribute to the unification of microbial ecology and macroecology.

## Author Contributions

RM designed all figures. AT-S built Table 1. AC built Tables 2 and 3. EM built Table 3. All authors listed co-authored and proof-read the manuscript. All authors approved the final manuscript.

## Funding

This work was primarily supported by the Serrapilheira Institute (grant number Serra-1709-17818). Further support was provided by the Coordenação de Aperfeiçoamento de Pessoal de Nível Superior—Brasil (CAPES)—Finance Code 001. AT-S thanks CAPES (88887.301758/2018-00). PMM thanks to PROPESQ-UFBA (11268). FC was supported by APOSTD/2018/186 Post-doctoral fellowship from Generalitat Valenciana. BD was supported by the Netherlands Organization for Scientific Research (NWO) Vidi grant 864.14.004. PG was supported by FAPESP (2017/08406-7) and CNPq. AC was supported by CNPq (132261/2018-9). RM was supported by CAPES (88882.460937/2019-01). AA was supported by São Paulo Research Foundation (FAPESP) grant number 2016/14277-2, and CAPES. RV was supported by CNPq, grant number 151157/2018-9.

## Conflict of Interest

The authors declare that the research was conducted in the absence of any commercial or financial relationships that could be construed as a potential conflict of interest.

## References

[B1] Aguilera P. A. Fernández A. Fernández R. Rumí R. Salmerón A. (2011). Bayesian networks in environmental modelling. Environ. Model. Sofftw. 26, 1376–1388. 10.1016/j.envsoft.2011.06.004

[B2] AIRS Science team Texeira J. (2008). Monthly CO2 in the free troposphere (AIRS-only) 2.5 degrees x 2 degrees V005 [Data set]. Goddard Earth Sci. Data Inf. Serv. Cent. (GES DISC). 10.5067/Aqua/AIRS/DATA336

[B3] Alameddine I. Cha Y. Reckhow K. H. (2011). An evaluation of automated structure learning with bayesian networks: an application to estuarine chlorophyll dynamics. Environ. Model. Soft. 26, 163–172. 10.1016/j.envsoft.2010.08.007

[B4] Altschul S. F. Gish W. Miller W. Myers E. W. Lipman D. J. (1990). Basic local alignment search tool. J. Mol. Biol. 215, 403–410. 10.1016/S0022-2836(05)80360-2 2231712

[B5] Amend A. S. Oliver T. A. Amaral-Zettler L. A. Boetius A. Fuhrman J. A. Horner-Devine M. C. (2013). Macroecological patterns of marine bacteria on a global scale. J. Biogeogr. 40, 800–811. 10.1111/jbi.12034

[B6] Anderson M. J. Ellingsen K. E. McArdle B. H. (2006). Multivariate dispersion as a measure of beta diversity. Ecol. Lett. 9, 683–693. 10.1111/j.1461-0248.2006.00926.x 16706913

[B7] Araújo M. B. Luoto M. (2007). The importance of biotic interactions for modelling species distributions under climate change. Glob. Ecol. Biogeogr. 16, 743–753. 10.1111/j.1466-8238.2007.00359.x

[B8] Araújo M. B. Pearson R. G. (2005). Equilibrium of species' distributions with climate. Ecography 28, 693–695. 10.1111/j.2005.0906-7590.04253.x

[B9] Araújo M. B. Rozenfeld A. (2013). The geographic scaling of biotic interactions. Ecography 6, no–no. 10.1111/j.1600-0587.2013.00643.x

[B10] Araújo M. B. Rozenfeld A. Rahbek C. Marquet P. A. (2011). Using species co-occurrence networks to assess the impacts of climate change. Ecography 34, 897–908. 10.1111/j.1600-0587.2011.06919.x

[B11] Astorga A. Oksanen J. Luoto M. Soininen J. Virtanen R. Muotka T. (2012). Distance decay of similarity in freshwater communities: do macro- and microorganisms follow the same rules? Glob. Ecol. Biogeogr. 21, 365–375. 10.1111/j.1466-8238.2011.00681.x

[B12] Ban Y. An L. Jiang H. (2015). Investigating microbial co-occurrence patterns based on metagenomic compositional data. Bioinformatics 31, 3322–3329. 10.1093/bioinformatics/btv364 26079350 PMC4795632

[B13] Banerjee S. Kirkby C. A. Schmutter D. Bissett A. Kirkegaard J. A. Richardson A. E. (2016). Network analysis reveals functional redundancy and keystone taxa amongst bacterial and fungal communities during organic matter decomposition in an arable soil. Soil Biol. Biochem. 97, 188–198. 10.1016/j.soilbio.2016.03.017

[B14] Banerjee S. Schlaeppi K. van der Heijden M. G. A. (2018). Keystone taxa as drivers of microbiome structure and functioning. Nat. Rev. Microbiol. 16, 567–576. 10.1038/s41579-018-0024-1 29789680

[B15] Barberán A. Bates S. T. Casamayor E. O. Fierer N. (2012). Using network analysis to explore co-occurrence patterns in soil microbial communities. ISME J. 6, 343–351. 10.1038/ismej.2011.119 21900968 PMC3260507

[B16] Barberán A. Casamayor E. O. Fierer N. (2014). The microbial contribution to macroecology. Front. Microbiol. 5, 203. 10.3389/fmicb.2014.00203 24829564 PMC4017162

[B17] Barberán A. Ladau J. Leff J. W. Pollard K. S. Menninger H. L. Dunn R. R. (2015). Continental-scale distributions of dust-associated bacteria and fungi. Proc. Natl. Acad. Sci. U. S. A. 112, 5756–5761. 10.1073/pnas.1420815112 25902536 PMC4426398

[B18] Barve N. Barve V. Jiménez-Valverde A. Lira-Noriega A. Maher S. P. Peterson A. T. (2011). The crucial role of the accessible area in ecological niche modeling and species distribution modeling. Ecol. Modell. 222, 1810–1819. 10.1016/j.ecolmodel.2011.02.011

[B19] Bastida F. García C. Fierer N. Eldridge D. J. Bowker M. A. Abades S. (2019). Global ecological predictors of the soil priming effect. Nat. Commun. 10, 3481. 10.1038/s41467-019-11472-7 31375717 PMC6677791

[B20] Bell T. (2010). Experimental tests of the bacterial distance–decay relationship. ISME J. 4, 1357. 10.1038/ismej.2010.77 20535220

[B21] Berry D. Widder S. (2014). Deciphering microbial interactions and detecting keystone species with co-occurrence networks. Front. Microbiol. 5, 219. 10.3389/fmicb.2014.00219 24904535 PMC4033041

[B22] Beuzen T. Marshall L. Splinter K. D. (2018). A comparison of methods for discretizing continuous variables in Bayesian Networks. Environ. Model. Software 108, 61–66.

[B23] Blaser M. J. Cardon Z. G. Cho M. K. Dangl J. L. Donohue T. J. Green J. L. (2016). Toward a Predictive Understanding of Earth's Microbiomes to Address 21st Century Challenges. MBio 7, 1–16. 10.1128/mBio.00714-16 PMC4895116 27178263

[B24] Bolger A. M. Lohse M. Usadel B. (2014). Trimmomatic: a flexible trimmer for Illumina sequence data. Bioinformatics 30, 2114–2120. 10.1093/bioinformatics/btu170 24695404 PMC4103590

[B25] Bond-Lamberty B. Bolton H. Fansler S. Heredia-Langner A. Liu C. McCue L. A. (2016). Soil respiration and bacterial structure and function after 17 years of a reciprocal soil transplant experiment. PloS One 11, e0150599. 10.1371/journal.pone.0150599 26934712 PMC4775055

[B26] Bovallius A. Roffey R. Henningson E. (1980). Long-range transmission of bacteria. Ann. N. Y. Acad. Sci. 353, 186–200. 10.1111/j.1749-6632.1980.tb18922.x 6939384

[B27] Bowman J. S. Ducklow H. W. (2015). Microbial Communities Can Be Described by Metabolic Structure: A General Framework and Application to a Seasonally Variable, Depth-Stratified Microbial Community from the Coastal West Antarctic Peninsula. PloS One 10, e0135868. 10.1371/journal.pone.0135868 26285202 PMC4540456

[B28] Bray J. R. Curtis J. T. (1957). An Ordination of the Upland Forest Communities of Southern Wisconsin. Ecol. Monogr. 27, 325–349. 10.2307/1942268

[B29] Breiman L. (2001). Random forest. Mach. Learn. 45, 5–32. 10.17849/insm-47-01-31-39.1

[B30] Brown S. M. Chen H. Hao Y. Laungani B. P. Ali T. A. Dong C. (2019). MGS-Fast: Metagenomic shotgun data fast annotation using microbial gene catalogs. GigaScience 8 (4), 1–9. 10.1093/gigascience/giz020 PMC6446249 30942867

[B31] Buchfink B. Xie C. Huson D. H. (2015). Fast and sensitive protein alignment using DIAMOND. Nat. Methods 12, 59–60. 10.1038/nmeth.3176 25402007

[B32] Caporaso J. G. Kuczynski J. Stombaugh J. Bittinger K. Bushman F. D. Costello E. K. (2010). QIIME allows analysis of high-throughput community sequencing data. Nat. Methods 7, 335–336. 10.1038/nmeth.f.303 20383131 PMC3156573

[B33] Cardona C. Weisenhorn P. Henry C. Gilbert J. A. (2016). Network-based metabolic analysis and microbial community modeling. Curr. Opin. Microbiol. 31, 124–131. 10.1016/j.mib.2016.03.008 27060776

[B34] Casanoves F. Pla L. Di Rienzo J. A. Díaz S. (2011). FDiversity: a software package for the integrated analysis of functional diversity. Methods Ecol. Evol. 2, 233–237. 10.1111/j.2041-210X.2010.00082.x

[B35] Chase J. M. Leibold M. A. (2002). Spatial scale dictates the productivity-biodiversity relationship. Nature 416, 427–430. 10.1038/416427a 11919631

[B36] Chen S. H. Pollino C. A. (2012). Good practice in Bayesian network modelling. Environ. Model. Softw. 37, 134–145. 10.1016/j.envsoft.2012.03.012

[B37] Clarke K. R. (1993). Non-parametric multivariate analyses of changes in community structure. Austral Ecol. 18, 117–143. 10.1111/j.1442-9993.1993.tb00438.x

[B38] Cohen W. B. Maiersperger T. K. Yang Z. Gower S. T. Turner D. P. Ritts W. D. (2003). Comparisons of land cover and LAI estimates derived from ETM+ and MODIS for four sites in North America: a quality assessment of 2000/2001 provisional MODIS products. Remote Sens. Environ. 88, 233–255. 10.1016/j.rse.2003.06.006

[B39] Cole J. R. Wang Q. Fish J. A. Chai B. McGarrell D. M. Sun Y. (2014). Ribosomal Database Project: data and tools for high throughput rRNA analysis. Nucleic Acids Res. 42, D633–D642. 10.1093/nar/gkt1244 24288368 PMC3965039

[B40] Colwell R. K. Rangel T. F. (2009). Hutchinson's duality: the once and future niche. Proc. Natl. Acad. Sci. U. S. A. 106 (Suppl 2), 19651–19658. 19805163 10.1073/pnas.0901650106 PMC2780946

[B41] Comte J. Lovejoy C. Crevecoeur S. Vincent W. F. (2016). Co-occurrence patterns in aquatic bacterial communities across changing permafrost landscapes. Biogeosciences 13, 175–190. 10.5194/bg-13-175-2016

[B42] Coyte K. Z. Schluter J. Foster K. R. (2015). The ecology of the microbiome: Networks, competition, and stability. Science 350, 663–666. 10.1126/science.aad2602 26542567

[B43] Crawley M. J. Harral J. E. (2001). Scale dependence in plant biodiversity. Science 291, 864–868. 10.1126/science.291.5505.864 11157164

[B44] de Araújo C. B. Marcondes-Machado L. O. Costa G. C. (2014). The importance of biotic interactions in species distribution models: a test of the Eltonian noise hypothesis using parrots. J. Biogeogr. 41, 513–523. 10.1111/jbi.12234

[B45] Death R. G. Death F. Stubbington R. Joy M. K. van den Belt M. (2015). How good are Bayesian belief networks for environmental management? A test with data from an agricultural river catchment. Freshw. Biol. 60, 2297–2309. 10.1111/fwb.12655

[B46] Debastiani V. J. Pillar V. D. (2012). SYNCSA—R tool for analysis of metacommunities based on functional traits and phylogeny of the community components. Bioinformatics 28, 2067–2068. 10.1093/bioinformatics/bts325 22668789

[B47] Delgado-Baquerizo M. Maestre F. T. Reich P. B. Trivedi P. Osanai Y. Liu Y. R. (2016). Carbon content and climate variability drive global soil bacterial diversity patterns. Ecol. Monograph. 86 (3), 373–390. 10.1002/ecm.1216/suppinfo

[B48] Delgado-Baquerizo M. Eldridge D. J. Maestre F. T. Karunaratne S. B. Trivedi P. Reich P. B. (2017). Climate legacies drive global soil carbon stocks in terrestrial ecosystems. Sci. Adv. 3, e1602008. 10.1126/sciadv.1602008 28439540 PMC5389782

[B49] Delgado-Baquerizo M. Oliverio A. M. Brewer T. E. Benavent-González A. Eldridge D. J. Bardgett R. D. (2018). A global atlas of the dominant bacteria found in soil. Science 359, 320–325. 10.1126/science.aap9516Z 29348236

[B50] DeSantis T. Z. Hugenholtz P. Larsen N. Rojas M. Brodie E. L. Keller K. (2006). Greengenes, a chimera-checked 16S rRNA gene database and workbench compatible with ARB. Appl. Environ. Microbiol. 72, 5069–5072. 10.1128/AEM.03006-05 16820507 PMC1489311

[B51] Devictor V. Mouillot D. Meynard C. Jiguet F. Thuiller W. Mouquet N. (2010). Spatial mismatch and congruence between taxonomic, phylogenetic and functional diversity: the need for integrative conservation strategies in a changing world. Ecol. Lett. 13, 1030–1040. 10.1111/j.1461-0248.2010.01493.x 20545736

[B52] Dıaz S. Cabido M. (2001). Vive la difference: plant functional diversity matters to ecosystem processes. Trends Ecol. Evol. 16, 646–655. 10.1016/S0169-5347(01)02283-2

[B53] Didan K. (2015). MOD13A3 MODIS/Terra vegetation Indices Monthly L3 Global 1km SIN Grid V006 [Data set]. NASA EOSDIS LP DAAC. 10.5067/MODIS/MOD13A3.006

[B54] Dinsdale E. A. Edwards R. A. Hall D. Angly F. Breitbart M. Brulc J. M. (2008). Functional metagenomic profiling of nine biomes. Nature 452, 629–632. 10.1038/nature06810 18337718

[B55] Drenovsky R. E. Steenwerth K. L. Jackson L. E. Scow K. M. (2010). Land use and climatic factors structure regional patterns in soil microbial communities. Glob. Ecol. Biogeogr. 19, 27–39. 10.1111/j.1466-8238.2009.00486.x 24443643 PMC3891896

[B56] Duarte L. da S. Carlucci M. B. Pillar V. D. (2009). Macroecological analyses reveal historical factors influencing seed dispersal strategies in Brazilian Araucaria forests. Glob. Ecol. Biogeogr. 18, 314–326. 10.1111/j.1466-8238.2009.00448.x

[B57] Elith J. Leathwick J. R. (2009). Species Distribution Models: Ecological Explanation and Prediction Across Space and Time. Annu. Rev. Ecol. Evol. Syst. 40, 677–697. 10.1146/annurev.ecolsys.110308.120159

[B58] Engelen R. J. Serrar S. Chevallier F. (2009). Four-dimensional data assimilation of atmospheric CO 2 using AIRS observations. J. Geophys. Res. 114, 631. 10.1029/2008JD010739

[B59] Fan K. Weisenhorn P. Gilbert J. A. Shi Y. Bai Y. Chu H. (2018). Soil pH correlates with the co-occurrence and assemblage process of diazotrophic communities in rhizosphere and bulk soils of wheat fields. Soil Biol. Biochem. 121, 185–192. 10.1016/j.soilbio.2018.03.017

[B60] Fang H. Huang C. Zhao H. Deng M. (2015). CCLasso: correlation inference for compositional data through Lasso. Bioinformatics 31, 3172–3180. 10.1093/bioinformatics/btv349 26048598 PMC4693003

[B61] Faure D. Joly D. (2016). “9 - Functional Ecology and Population Genomics,” in Insight on Environmental Genomics. Eds. Faure D. Joly D. (Amsterdam, Netherlands: Elsevier), 93–102.

[B62] Faust K. Raes J. (2012). Microbial interactions: from networks to models. Nat. Rev. Microbiol. 10, 538–550. 10.1038/nrmicro2832 22796884

[B63] Faust K. Raes J. (2016). CoNet app: inference of biological association networks using Cytoscape. F1000Res 5, 1519. 10.12688/f1000research.9050.2 27853510 PMC5089131

[B64] Fenchel T. Finlay B. J. (2004). The Ubiquity of Small Species: Patterns of Local and Global Diversity. Bioscience 54, 777–784. 10.1641/0006-3568(2004)054[0777:tuossp]2.0.co

[B65] Fick S. E. Hijmans R. J. (2017). WorldClim 2: new 1-km spatial resolution climate surfaces for global land areas: NEW CLIMATE SURFACES FOR GLOBAL LAND AREAS. Int. J. Climatol. 37, 4302–4315. 10.1002/joc.5086

[B66] Fierer N. Jackson R. B. (2006). The diversity and biogeography of soil bacterial communities. Proc. Natl. Acad. Sci. U. S. A. 103, 626–631. 10.1073/pnas.0507535103 16407148 PMC1334650

[B67] Fierer N. McCain C. M. Meir P. Zimmermann M. Rapp J. M. Silman M. R. (2011). Microbes do not follow the elevational diversity patterns of plants and animals. Ecology 92, 797–804. 10.1890/10-1170.1 21661542

[B68] Finlay B. J. Clarke K. J. (1999). Ubiquitous dispersal of microbial species. Nature 400, 828–828. 10.1038/23616

[B69] Friedl M. Sulla-Menashe D. (2015). MCD12C1 MODIS/Terra+Aqua Land Cover Type Yearly L3 Global 0.05Deg CMG V006 [Data set]. NASA EOSDIS L. Process. DAAC. 10.5067/MODIS/MCD12C1.006

[B70] Friedman J. Alm E. J. (2012). Inferring correlation networks from genomic survey data. PloS Comput. Biol. 8, e1002687. 10.1371/journal.pcbi.1002687 23028285 PMC3447976

[B71] Fu H. Zhong J. Fang S. Hu J. Guo C. Lou Q. (2017). Scale-dependent changes in the functional diversity of macrophytes in subtropical freshwater lakes in south China. Sci. Rep. 7, 8294. 10.1038/s41598-017-08844-8 28811648 PMC5557923

[B72] Fuhrman J. A. Steele J. A. Hewson I. Schwalbach M. S. Brown M. V. Green J. L. (2008). A latitudinal diversity gradient in planktonic marine bacteria. Proc. Natl. Acad. Sci. U. S. A. 105, 7774–7778. 10.1073/pnas.0803070105 18509059 PMC2409396

[B73] Galand P. E. Pereira O. Hochart C. Auguet J. C. Debroas D. (2018). A strong link between marine microbial community composition and function challenges the idea of functional redundancy. ISME J. 12, 2470–2478. 10.1038/s41396-018-0158-1 29925880 PMC6155072

[B74] Giglio L. Justice C. Boschetti L. Roy D. (2015). MCD64A1 MODIS/Terra+Aqua Burned Area Monthly L3 Global 500m SIN Grid V006 [Data set]. NASA EOSDIS L. Process. DAAC. 10.5067/MODIS/MCD64A1.006

[B75] Gotelli N. J. Graves G. R. Rahbek C. (2010). Macroecological signals of species interactions in the Danish avifauna. Proc. Natl. Acad. Sci. U. S. A. 107, 5030–5035. 10.1073/pnas.0914089107 20194760 PMC2841898

[B76] Grace J. B. (2006). Structural equation modeling natural systems (Cambridge, UK: Cambridge University Press).

[B77] Grenié M. Denelle P. Tucker C. M. Munoz F. Violle C. (2017). funrar: An R package to characterize functional rarity. Divers. Distrib. 23, 1365–1371. 10.1111/ddi.12629

[B78] Guimarães P. R. Jr. Pires M. M. Jordano P. Bascompte J. Thompson J. N. (2017). Indirect effects drive coevolution in mutualistic networks. Nature 550, 511–514. 10.1038/nature24273 29045396

[B79] Guimera R. Amaral L. A. N. (2005). Functional cartography of complex metabolic networks. Nature 433, 895–900. 10.1038/nature03288 15729348 PMC2175124

[B80] Guisan A. Rahbek C. (2011). SESAM - a new framework integrating macroecological and species distribution models for predicting spatio-temporal patterns of species assemblages. J. Biogeogr. 38, 1433–1444. 10.1111/j.1365-2699.2011.02550.x

[B81] Hampton S. E. Strasser C. A. Tewksbury J. J. Gram W. K. Budden A. E. Batcheller A. L. (2013). Big data and the future of ecology. Front. Ecol. Environ. 11, 156–162. 10.1890/120103

[B82] Hanson C. A. Fuhrman J. A. Horner-Devine M. C. Martiny J. B. H. (2012). Beyond biogeographic patterns: processes shaping the microbial landscape. Nat. Rev. Microbiol. 10, 497–506. 10.1038/nrmicro2795 22580365

[B83] Harris D. J. (2015). Generating realistic assemblages with a joint species distribution model. Methods Ecol. Evol. 6, 465–473. 10.1111/2041-210X.12332

[B84] Hartemink A. J. (2001). Principled computational methods for the validation discovery of genetic regulatory networks, (Doctoral dissertation, Massachusetts Institute of Technology). Available at: https://dspace.mit.edu/handle/1721.1/8699?show=full [Accessed August 19, 2019].

[B85] Hartman K. van der Heijden M. G. A. Wittwer R. A. Banerjee S. Walser J.-C. Schlaeppi K. (2018). Cropping practices manipulate abundance patterns of root and soil microbiome members paving the way to smart farming. Microbiome 6, 14. 10.1186/s40168-017-0389-9 29338764 PMC5771023

[B86] Hatzenpichler R. (2012). Diversity, physiology, and niche differentiation of ammonia-oxidizing archaea. Appl. Environ. Microbiol. 78, 7501–7510. 10.1128/AEM.01960-12 22923400 PMC3485721

[B87] Hendershot J. N. Read Q. D. Henning J. A. Sanders N. J. Classen A. T. (2017). Consistently inconsistent drivers of microbial diversity and abundance at macroecological scales. Ecology 98, 1757–1763. 10.1002/ecy.1829 28380683

[B88] Hengl T. Mendes de Jesus J. Heuvelink G. B. M. Ruiperez Gonzalez M. Kilibarda M. Blagotić A. (2017). SoilGrids250m: Global gridded soil information based on machine learning. PLoS One 12, e0169748. 10.1371/journal.pone.0169748 28207752 PMC5313206

[B89] Hijmans R. J. Phillips S. Leathwick J. Elith J. Hijmans M. R. J. (2017). Package ‘dismo.'. Circles 9, 1–68. 10.1002/joc.5086

[B90] Hillebrand H. (2004). On the generality of the latitudinal diversity gradient. Am. Nat. 163, 192–211. 282400/381004 14970922 10.1086/381004

[B91] Hong Y. Hsu K.-L. Sorooshian S. Gao X. (2004). Precipitation Estimation from Remotely Sensed Imagery Using an Artificial Neural Network Cloud Classification System. J. Appl. Meteorol. 43, 1834–1853. 10.1175/JAM2173.1

[B92] Holt R. D. (2009). Bringing the Hutchinsonian niche into the 21st century: ecological and evolutionary perspectives. Proc. Natl. Acad. Sci. U. S. A. 106 Suppl 2, 19659–19665. 10.1073/pnas.0905137106 19903876 PMC2780934

[B93] Horner-Devine M. C. Lage M. Hughes J. B. Bohannan B. J. M. (2004). A taxa-area relationship for bacteria. Nature 432, 750–753. 10.1038/nature03073 15592412

[B94] Horner-Devine M. C. Silver J. M. Leibold M. A. Bohannan B. J. M. Colwell R. K. Fuhrman J. A. (2007). A comparison of taxon co-occurrence patterns for macro- and microorganisms. Ecology 88, 1345–1353. 10.1890/06-0286 17601127

[B95] Hortal J. de Bello F. Diniz-Filho J. A. F. Lewinsohn T. M. Lobo J. M. Ladle R. J. (2015). Seven Shortfalls that Beset Large-Scale Knowledge of Biodiversity. Annu. Rev. Ecol. Evol. Syst. 46, 523–549. 10.1146/annurev-ecolsys-112414-054400

[B96] Huffman G. J. Bolvin D. T. Nelkin E. J. Wolff D. B. Adler R. F. Gu G. (2007). The TRMM Multisatellite Precipitation Analysis (TMPA): Quasi-Global, Multiyear, Combined-Sensor Precipitation Estimates at Fine Scales. J. Hydrometeorol. 8, 38–55. 10.1175/JHM560.1

[B97] Hug L. A. Baker B. J. Anantharaman K. Brown C. T. Probst A. J. Castelle C. J. (2016a). A new view of the tree of life. Nat. Microbiol. 1, 16048. 10.1038/nmicrobiol.2016.48 27572647

[B98] Hug L. A. Thomas B. C. Sharon I. Brown C. T. Sharma R. Hettich R. L. (2016b). Critical biogeochemical functions in the subsurface are associated with bacteria from new phyla and little studied lineages. Environ. Microbiol. 18, 159–173. 10.1111/1462-2920.12930 26033198

[B99] Hugenholtz P. Tyson G. W. (2008). Microbiology: metagenomics. Nature 455, 481–483. 10.1038/455481a 18818648

[B100] Huson D. H. Albrecht B. Bağcı C. Bessarab I. Górska A. Jolic D. (2018). MEGAN-LR: new algorithms allow accurate binning and easy interactive exploration of metagenomic long reads and contigs. Biol. Direct 13, 6. 10.1186/s13062-018-0208-7 29678199 PMC5910613

[B101] Hutchins D. A. Mulholland M. R. Fu F. (2009). Nutrient Cycles and Marine Microbes in a CO₂-Enriched Ocean. Oceanography 22, 128–145. 10.5670/oceanog.2009.103

[B102] Jackson M. A. Bonder M. J. Kuncheva Z. Zierer J. Fu J. Kurilshikov A. (2018). Detection of stable community structures within gut microbiota co-occurrence networks from different human populations. PeerJ 6, e4303. 10.7717/peerj.4303 29441232 PMC5807925

[B103] Jarzyna M. A. Jetz W. (2018). Taxonomic and functional diversity change is scale dependent. Nat. Commun. 9, 2565. 10.1038/s41467-018-04889-z 29967400 PMC6028399

[B104] Jay C. V. Marcot B. G. Douglas D. C. (2011). Projected status of the Pacific walrus (Odobenus rosmarus divergens) in the twenty-first century. Polar Biol. 34, 1065–1084. 10.1007/s00300-011-0967-4

[B105] Jessup C. M. Kassen R. Forde S. E. Kerr B. Buckling A. Rainey P. B. (2004). Big questions, small worlds: microbial model systems in ecology. Trends Ecol. Evol. 19, 189–197. 10.1016/j.tree.2004.01.008 16701253

[B106] Jiao S. Liu Z. Lin Y. Yang J. Chen W. Wei G. (2016). Bacterial communities in oil contaminated soils: Biogeography and co-occurrence patterns. Soil Biol. Biochem. 98, 64–73. 10.1016/j.soilbio.2016.04.005

[B107] Jost L. (2007). Partitioning diversity into independent alpha and beta components. Ecology 88, 2427–2439. 10.1890/06-1736.1 18027744

[B108] Kass J. M. Vilela B. Aiello-Lammens M. E. Muscarella R. Merow C. Anderson R. P. (2018). Wallace: a flexible platform for reproducible modeling of species niches and distributions built for community expansion. Methods Ecol. Evol. 9, 1151–1156. 10.1111/2041-210X.12945

[B109] Kearney M. Porter W. (2009). Mechanistic niche modelling: combining physiological and spatial data to predict species' ranges. Ecol. Lett. 12, 334–350. 10.1111/j.1461-0248.2008.01277.x 19292794

[B110] Keller C. K. White T. M. O'brien R. Smith J. L. (2006). Soil CO2 dynamics and fluxes as affected by tree harvest in an experimental sand ecosystem. J. Geophys. Res.: Biogeosci. 111, (G3). 10.1029/2005jg000157

[B111] Kerr J. T. Kharouba H. M. Currie D. J. (2007). The macroecological contribution to global change solutions. Science 316, 1581–1584. 10.1126/science.1133267 17569854

[B112] Koslicki D. Falush D. (2016). MetaPalette: a k-mer Painting Approach for Metagenomic Taxonomic Profiling and Quantification of Novel Strain Variation. mSystems 1, 1–18. 10.1128/mSystems.00020-16 PMC5069763 27822531

[B113] Koza J. R. Bennett F. H., III Andre D. Keane M. A. (2000). Synthesis of topology and sizing of analog electrical circuits by means of genetic programming. Comput. Methods Appl. Mech. Eng. 186, 459–482. 10.1109/4235.687879

[B114] Koza J. R. (1992). Genetic Programming: On the Programming of Computers by Means of Natural Selection (Cambridge, Massachusetts, USA: MIT Press).

[B115] Kultima J. R. Coelho L. P. Forslund K. Huerta-Cepas J. Li S. S. Driessen M. (2016). MOCAT2: a metagenomic assembly, annotation and profiling framework. Bioinformatics 32, 2520–2523. 10.1093/bioinformatics/btw183 27153620 PMC4978931

[B116] Kumar S. V. Peters-Lidard C. D. Tian Y. Houser P. R. Geiger J. Olden S. (2006). Land information system: An interoperable framework for high resolution land surface modeling. Environ. Model. Softw. 21, 1402–1415. 10.1016/j.envsoft.2005.07.004

[B117] Kurtz Z. D. Müller C. L. Miraldi E. R. Littman D. R. Blaser M. J. Bonneau R. A. (2015). Sparse and compositionally robust inference of microbial ecological networks. PloS Comput. Biol. 11, e1004226. 10.1371/journal.pcbi.1004226 25950956 PMC4423992

[B118] Laliberté E. Legendre P. (2010). A distance-based framework for measuring functional diversity from multiple traits. Ecology 91, 299–305. 20380219 10.1890/08-2244.1

[B119] Langille M. G. I. Zaneveld J. Caporaso J. G. McDonald D. Knights D. Reyes J. A. (2013). Predictive functional profiling of microbial communities using 16S rRNA marker gene sequences. Nat. Biotechnol. 31, 814–821. 10.1038/nbt.2676 23975157 PMC3819121

[B120] Larsen P. E. Field D. Gilbert J. A. (2012). Predicting bacterial community assemblages using an artificial neural network approach. Nat. Methods 9, 621–625. 10.1038/nmeth.1975 22504588

[B121] Lauber C. L. Hamady M. Knight R. Fierer N. (2009). Pyrosequencing-based assessment of soil pH as a predictor of soil bacterial community structure at the continental scale. Appl. Environ. Microbiol. 75, 5111–5120. 10.1128/AEM.00335-09 19502440 PMC2725504

[B122] Layeghifard M. Hwang D. M. Guttman D. S. (2017). Disentangling Interactions in the Microbiome: A Network Perspective. Trends Microbiol. 25, 217–228. 10.1016/j.tim.2016.11.008 27916383 PMC7172547

[B123] Legendre P. Legendre L. F. J. (2012). Numerical Ecology. (Elsevier Amsterdam, Netherlands).

[B124] Legendre P. Borcard D. Peres-Neto P. R. (2005). Analyzing beta diversity: partitioning the spatial variation of community composition data. Ecol. Monogr. 75, 435–450. 10.1890/05-0549

[B125] Leinonen R. Akhtar R. Birney E. Bower L. Cerdeno-Tárraga A. Cheng Y. (2011a). The European Nucleotide Archive. Nucleic Acids Res. 39, D28–D31. 10.1093/nar/gkq967 20972220 PMC3013801

[B126] Leinonen R. Sugawara H. Shumway M. International Nucleotide Sequence Database Collaboration (2011b). The sequence read archive. Nucleic Acids Res. 39, D19–D21. 10.1093/nar/gkq1019 21062823 PMC3013647

[B127] Levin S. A. (1992). The Problem of Pattern and Scale in Ecology: The Robert H. MacArthur Award Lecture. Ecology 73, 1943–1967. 10.2307/1941447

[B128] Li D. Zhan M. Liu H. Liao Y. Liao G. (2017). A Robust Translational Motion Compensation Method for ISAR Imaging Based on Keystone Transform and Fractional Fourier Transform Under Low SNR Environment. IEEE Trans. Aerosp. Electron. Syst. 53, 2140–2156. 10.1109/TAES.2017.2683599

[B129] Liaw A. Wiener M. (2002). Classification and Regression by randomForest. R News 2 (3), 18–22.

[B130] Lima-Mendez G. Faust K. Henry N. Decelle J. (2015). Determinants of community structure in the global plankton interactome. Science 348 (6237), 1262073. 25999517 10.1126/science.1262073

[B131] Lin H.-H. Liao Y.-C. (2016). Accurate binning of metagenomic contigs *via* automated clustering sequences using information of genomic signatures and marker genes. Sci. Rep. 6, 24175. 10.1038/srep24175 27067514 PMC4828714

[B132] Lin H. Yu B. Chen Z. Hu Y. Huang Y. Wu J. (2013). A geospatial web portal for sharing and analyzing greenhouse gas data derived from satellite remote sensing images. Front. Earth Sci. 7, 295–309. 10.1007/s11707-013-0365-z

[B133] Lomolino M. V. (2001). Elevation gradients of species-density: historical and prospective views. Glob. Ecol. Biogeogr. 10, 3–13. 10.1046/j.1466-822x.2001.00229.x

[B134] Louca S. Parfrey L. W. Doebeli M. (2016). Decoupling function and taxonomy in the global ocean microbiome. Science 353, 1272–1277. 10.1126/science.aaf4507 27634532

[B135] Louca S. Polz M. F. Mazel F. Albright M. B. N. Huber J. A. O'Connor M. I. (2018). Function and functional redundancy in microbial systems. Nat. Ecol. Evol. 2, 936–943. 10.1038/s41559-018-0519-1 29662222

[B136] Lupatini M. Suleiman A. K. A. Jacques R. J. S. Antoniolli Z. I. de Siqueira Ferreira A. Kuramae E. E. (2014). Network topology reveals high connectance levels and few key microbial genera within soils. Front. Environ. Sci. Eng. China 2, 343. 10.3389/fenvs.2014.00010

[B137] Mace G. M. Norris K. Fitter A. H. (2012). Biodiversity and ecosystem services: a multilayered relationship. Trends Ecol. Evol. 27, 19–26. 10.1016/j.tree.2011.08.006 21943703

[B138] Maddy E. S. Barnet C. D. Goldberg M. Sweeney C. Liu X. (2008). CO2 retrievals from the Atmospheric Infrared Sounder: Methodology and validation. J. Geophys. Res. D: Atmos. 113, (D11). 10.1029/2007jd009402

[B139] Marasco R. Rolli E. Fusi M. Michoud G. Daffonchio D. (2018). Grapevine rootstocks shape underground bacterial microbiome and networking but not potential functionality. Microbiome 6, 3. 10.1186/s40168-017-0391-2 29298729 PMC5751889

[B140] Martiny J. B. H. Bohannan B. J. M. Brown J. H. Colwell R. K. Fuhrman J. A. Green J. L. (2006). Microbial biogeography: putting microorganisms on the map. Nat. Rev. Microbiol. 4, 102–112. 10.1038/nrmicro1341 16415926

[B141] May R. M. (1972). Will a large complex system be stable? Nature 238, 413–414. 10.1038/238413a0 4559589

[B142] McDonald D. Price M. N. Goodrich J. Nawrocki E. P. DeSantis T. Z. Probst A. (2012). An improved Greengenes taxonomy with explicit ranks for ecological and evolutionary analyses of bacteria and archaea. ISME J. 6, 610–618. 10.1038/ismej.2011.139 22134646 PMC3280142

[B143] McGill B. J. Nekola J. C. (2010). Mechanisms in macroecology: AWOL or purloined letter? Towards a pragmatic view of mechanism. Oikos 119, 591–603. 10.1111/j.1600-0706.2009.17771.x

[B144] McGill B. (2003). Strong and weak tests of macroecological theory. Oikos 102, 679–685. 10.1034/j.1600-0706.2003.12617.x

[B145] McGill B. J. (2010). Ecology. Matters of scale. Science 328, 575–576. 10.1126/science.1188528 20431001

[B146] Mendes L. W. Mendes R. Raaijmakers J. M. Tsai S. M. (2018). Breeding for soil-borne pathogen resistance impacts active rhizosphere microbiome of common bean. ISME J. 12, 3038–3042. 10.1038/s41396-018-0234-6 30018368 PMC6246553

[B147] Meyer F. Paarmann D. D'Souza M. Olson R. Glass E. M. Kubal M. (2008). The metagenomics RAST server - a public resource for the automatic phylogenetic and functional analysis of metagenomes. BMC Bioinf. 9, 386. 10.1186/1471-2105-9-386 PMC2563014 18803844

[B148] Mitchell A. L. Scheremetjew M. Denise H. Potter S. Tarkowska A. Qureshi M. (2018). EBI Metagenomics in 2017: enriching the analysis of microbial communities, from sequence reads to assemblies. Nucleic Acids Res. 46, D726–D735. 10.1093/nar/gkx967 29069476 PMC5753268

[B149] Morgan C. G. Allen M. Liang M. C. Shia R. L. Blake G. A. Yung Y. L. (2004). Isotopic fractionation of nitrous oxide in the stratosphere: Comparison between model and observations. J. Geophys. Res. D: Atmos. 109, (D4) 10.1029/2003jd003402

[B150] Mouchet M. A. Villéger S. Mason N. W. H. Mouillot D. (2010). Functional diversity measures: an overview of their redundancy and their ability to discriminate community assembly rules. Funct. Ecol. 24, 867–876. 10.1111/j.1365-2435.2010.01695.x

[B151] Nabout J. C. Caetano J. M. Ferreira R. B. Teixeira I. R. de Freitas Alves S. M. (2012). Using Correlative, Mechanistic and Hybrid Niche Models to Predict the Productivity and Impact of Global Climate Change on Maize Crop in Brazil. Natureza Conservação 10, 177–183. 10.4322/natcon.2012.034

[B152] Naimi B. Araújo M. B. (2016). sdm: a reproducible and extensible R platform for species distribution modelling. Ecography 39, 368–375. 10.1111/ecog.01881

[B153] Nelson M. B. Martiny A. C. Martiny J. B. H. (2016). Global biogeography of microbial nitrogen-cycling traits in soil. Proc. Natl. Acad. Sci. U. S. A. 113, 8033–8040. 10.1073/pnas.1601070113 27432978 PMC4961168

[B154] Nogués-Bravo D. (2009). Predicting the past distribution of species climatic niches. Glob. Ecol. Biogeogr. 18, 521–531. 10.1111/j.1466-8238.2009.00476.x

[B155] Noguchi H. Park J. Takagi T. (2006). MetaGene: prokaryotic gene finding from environmental genome shotgun sequences. Nucleic Acids Res. 34, 5623–5630. 10.1093/nar/gkl723 17028096 PMC1636498

[B156] Noguez A. M. Arita H. T. Escalante A. E. Forney L. J. García-Oliva F. Souza V. (2005). Microbial macroecology: highly structured prokaryotic soil assemblages in a tropical deciduous forest. Glob. Ecol. Biogeogr. 14, 241–248. 10.1111/j.1466-822X.2005.00156.x

[B157] Nojavan A. F. Qian S. S. Paerl H. W. Reckhow K. H. Albright E. A. (2014). A study of anthropogenic and climatic disturbance of the New River Estuary using a Bayesian belief network. Mar. Pollut. Bull. 83, 107–115. 10.1016/j.marpolbul.2014.04.011 24814252

[B158] Nojavan A. F. Qian S. S. Stow C. A. (2017). Comparative analysis of discretization methods in Bayesian networks. Environ. Model. Softw. 87, 64–71. 10.1016/j.envsoft.2016.10.007

[B159] Nottingham A. T. Fierer N. Turner B. L. Whitaker J. Ostle N. J. McNamara N. P. (2018). Microbes follow Humboldt: temperature drives plant and soil microbial diversity patterns from the Amazon to the Andes. Ecology 99, 2455–2466. 10.1002/ecy.2482 30076592 PMC6850070

[B160] Ofori B. Y. Stow A. J. Baumgartner J. B. Beaumont L. J. (2017). Influence of adaptive capacity on the outcome of climate change vulnerability assessment. Sci. Rep. 7, 12979. 10.1038/s41598-017-13245-y 29021590 PMC5636830

[B161] Ohgushi T. (2005). Indirect Interaction Webs: Herbivore-Induced Effects Through Trait Change in Plants. Annu. Rev. Ecol. Evol. Syst. 36, 81–105. 10.1146/annurev.ecolsys.36.091704.175523

[B162] Oliver T. H. Heard M. S. Isaac N. J. B. Roy D. B. Procter D. Eigenbrod F. (2015). Biodiversity and Resilience of Ecosystem Functions. Trends Ecol. Evol. 30, 673–684. 10.1016/j.tree.2015.08.009 26437633

[B163] Ounit R. Wanamaker S. Close T. J. Lonardi S. (2015). CLARK: fast and accurate classification of metagenomic and genomic sequences using discriminative k-mers. BMC Genomics 16, 236. 10.1186/s12864-015-1419-2 25879410 PMC4428112

[B164] Paine R. T. (1966). Food Web Complexity and Species Diversity. Am. Nat. 100, 65–75. 282400/282400

[B165] Paine R. T. (1969). The Pisaster-Tegula interaction: prey patches, predator food preference, and intertidal community structure. Ecology 50, 950–961. 10.2307/1936888

[B166] Pearl J. (2014). Probabilistic Reasoning in Intelligent Systems: Networks of Plausible Inference. (Elsevier, Amsterdam, Netherlands).

[B167] Peay K. G. Garbelotto M. Bruns T. D. (2010). Evidence of dispersal limitation in soil microorganisms: isolation reduces species richness on mycorrhizal tree islands. Ecology 91, 3631–3640. 10.1890/09-2237.1 21302834

[B168] Pecl G. T. Araújo M. B. Bell J. D. Blanchard J. Bonebrake T. C. Chen I.-C. (2017). Biodiversity redistribution under climate change: Impacts on ecosystems and human well-being. Science 355, 1–9. 10.1126/science.aai9214 28360268

[B169] Petchey O. L. Gaston K. J. (2002). Functional diversity (FD), species richness and community composition. Ecol. Lett. 5, 402–411. 10.1046/j.1461-0248.2002.00339.x

[B170] Petchey O. L. Gaston K. J. (2006). Functional diversity: back to basics and looking forward. Ecol. Lett. 9, 741–758. 10.1111/j.1461-0248.2006.00924.x 16706917

[B171] Petchey O. L. Hector A. Gaston K. J. (2004). How do different measures of functional diversity perform? Ecology 85, 847–857. 10.1890/03-0226

[B172] Peters-Lidard C. D. Houser P. R. Tian Y. Kumar S. V. Geiger J. Olden S. (2007). High-performance Earth system modeling with NASA/GSFC's Land Information System. Innov. Syst. Software Eng. 3, 157–165. 10.1007/s11334-007-0028-x

[B173] Peterson A. T. Knapp S. Guralnick R. Soberón J. Holder M. T. (2010). The big questions for biodiversity informatics. Syst. Biodivers. 8, 159–168. 10.1080/14772001003739369

[B174] Phan T. D. Smart J. C. R. Capon S. J. Hadwen W. L. Sahin O. (2016). Applications of Bayesian belief networks in water resource management: A systematic review. Environ. Model. Softw. 85, 98–111. 10.1016/j.envsoft.2016.08.006

[B175] Phillips S. J. Dudík M. (2008). Modeling of species distributions with Maxent: new extensions and a comprehensive evaluation. Ecography 31, 161–175. 10.1111/j.0906-7590.2008.5203.x

[B176] Pollock L. J. Tingley R. Morris W. K. Golding N. O'Hara R. B. Parris K. M. (2014). Understanding co-occurrence by modelling species simultaneously with a Joint Species Distribution Model (JSDM). Methods Ecol. Evol. 5, 397–406. 10.1111/2041-210X.12180

[B177] Poudel R. Jumpponen A. Schlatter D. C. Paulitz T. C. Gardener B. B. M. Kinkel L. L. (2016). Microbiome Networks: A Systems Framework for Identifying Candidate Microbial Assemblages for Disease Management. Phytopathology 106, 1083–1096. 10.1094/PHYTO-02-16-0058-FI 27482625

[B178] Qian S. S. Miltner R. J. (2015). A continuous variable Bayesian networks model for water quality modeling: A case study of setting nitrogen criterion for small rivers and streams in Ohio, USA. Environ. Model. Softw. 69, 14–22. 10.1016/j.envsoft.2015.03.001

[B179] Quast C. Pruesse E. Yilmaz P. Gerken J. Schweer T. Yarza P. (2013). The SILVA ribosomal RNA gene database project: improved data processing and web-based tools. Nucleic Acids Res. 41, D590–D596. 10.1093/nar/gks1219 23193283 PMC3531112

[B180] Rahbek C. (2005). The role of spatial scale and the perception of large-scale species-richness patterns. Ecol. Lett. 8, 224–239. 10.1111/j.1461-0248.2004.00701.x

[B181] Ramirez K. S. Döring M. Eisenhauer N. Gardi C. Ladau J. Leff J. W. (2015). Toward a global platform for linking soil biodiversity data. Front. Ecol. Evol. 3, 2189. 10.3389/fevo.2015.00091

[B182] Ramirez K. S. Knight C. G. de Hollander M. Brearley F. Q. Constantinides B. Cotton A. (2018). Detecting macroecological patterns in bacterial communities across independent studies of global soils. Nat. Microbiol. 3, 189–196. 10.1038/s41564-017-0062-x 29158606

[B183] Ren Z. Wang F. Qu X. Elser J. J. Liu Y. Chu L. (2017). Taxonomic and Functional Differences between Microbial Communities in Qinghai Lake and Its Input Streams. Front. Microbiol. 8, 2319. 10.3389/fmicb.2017.02319 29213266 PMC5702853

[B184] Ricotta C. de Bello F. Moretti M. Caccianiga M. Cerabolini B. E. L. Pavoine S. (2016). Measuring the functional redundancy of biological communities: a quantitative guide. Methods Ecol. Evol. 7, 1386–1395. 10.1111/2041-210X.12604

[B185] Riesenfeld C. S. Schloss P. D. Handelsman J. (2004). Metagenomics: genomic analysis of microbial communities. Annu. Rev. Genet. 38, 525–552. 10.1146/annurev.genet.38.072902.091216 15568985

[B186] Rodell M. Houser P. R. Jambor U. Gottschalck J. Mitchell K. Meng C.-J. (2004). The Global Land Data Assimilation System. Bull. Am. Meteorol. Soc 85, 381–394. 10.1175/BAMS-85-3-381

[B187] Rodell M. Velicogna I. Famiglietti J. S. (2009). Satellite-based estimates of groundwater depletion in India. Nature 460, 999–1002. 10.1038/nature08238 19675570

[B188] Running S. Mu Q. Zhao M. (2017). MOD16A3 MODIS/Terra Net Evapotranspiration Yearly L4 Global 500m SIN Grid V006 [Data set]. NASA EOSDIS L. Process. DAAC. 10.5067/MODIS/MOD16A3.006

[B189] Safi K. Cianciaruso M. V. Loyola R. D. Brito D. Armour-Marshall K. Diniz-Filho J. A. F. (2011). Understanding global patterns of mammalian functional and phylogenetic diversity. Philos. Trans. R. Soc Lond. B Biol. Sci. 366, 2536–2544. 10.1098/rstb.2011.0024 21807734 PMC3138614

[B190] Savtchenko A. Ouzounov D. Ahmad S. Acker J. Leptoukh G. Koziana J. (2004). Terra and Aqua MODIS products available from NASA GES DAAC. Adv. Sp. Res. 34, 710–714. 10.1016/j.asr.2004.03.012

[B191] Scher J. U. Bretz W. A. Abramson S. B. (2014). Periodontal disease and subgingival microbiota as contributors for rheumatoid arthritis pathogenesis: modifiable risk factors? Curr. Opin. Rheumatol. 26, 424–429. 10.1097/BOR.0000000000000076 24807405 PMC4128331

[B192] Schleuter D. Daufresne M. Massol F. Argillier C. (2010). A user's guide to functional diversity indices. Ecol. Monogr. 80, 469–484. 10.1890/08-2225.1

[B193] Schmidt T. M. DeLong E. F. Pace N. R. (1991). Analysis of a marine picoplankton community by 16S rRNA gene cloning and sequencing. J. Bacteriol. 173, 4371–4378. 10.1128/jb.173.14.4371-4378.1991 2066334 PMC208098

[B194] Schmieder R. Edwards R. (2011). Quality control and preprocessing of metagenomic datasets. Bioinformatics 27, 863–864. 10.1093/bioinformatics/btr026 21278185 PMC3051327

[B195] Sczyrba A. Hofmann P. Belmann P. Koslicki D. Janssen S. Dröge J. (2017). Critical assessment of metagenome interpretation—a benchmark of metagenomics software. Nat. Methods 14, 1063. 10.1038/nmeth.4458 28967888 PMC5903868

[B196] Shade A. Dunn R. R. Blowes S. A. Keil P. Bohannan B. J. M. Herrmann M. (2018). Macroecology to Unite All Life, Large and Small. Trends Ecol. Evol. 33, 731–744. 10.1016/j.tree.2018.08.005 30209011

[B197] Shannon P. Markiel A. Ozier O. Baliga N. S. Wang J. T. Ramage D. (2003). Cytoscape: a software environment for integrated models of biomolecular interaction networks. Genome Res. 13, 2498–2504. 10.1101/gr.1239303 14597658 PMC403769

[B198] Silva D. P. Vilela B. De Marco P. Jr. Nemésio A. (2014). Using ecological niche models and niche analyses to understand speciation patterns: the case of sister neotropical orchid bees. PloS One 9, e113246. 10.1371/journal.pone.0113246 25422941 PMC4244149

[B199] Silva D. P. Vilela B. Buzatto B. A. Moczek A. P. Hortal J. (2016a). Contextualized niche shifts upon independent invasions by the dung beetle Onthophagus taurus. Biol. Invasions 18, 3137–3148. 10.1007/s10530-016-1204-4

[B200] Silva G. G. Z. Green K. T. Dutilh B. E. Edwards R. A. (2016b). SUPER-FOCUS: a tool for agile functional analysis of shotgun metagenomic data. Bioinformatics 32, 354–361. 10.1093/bioinformatics/btv584 26454280 PMC4734042

[B201] Smolik M. G. Dullinger S. Essl F. Kleinbauer I. Leitner M. Peterseil J. (2010). Integrating species distribution models and interacting particle systems to predict the spread of an invasive alien plant. J. Biogeogr. 37, 411–422. 10.1111/j.1365-2699.2009.02227.x

[B202] Snyder L. A. S. Loman N. Pallen M. J. Penn C. W. (2009). Next-generation sequencing–the promise and perils of charting the great microbial unknown. Microb. Ecol. 57, 1–3. 10.1007/s00248-008-9465-9 19015912

[B203] Soberón J. Nakamura M. (2009). Niches and distributional areas: concepts, methods, and assumptions. Proc. Natl. Acad. Sci. U. S. A. 106 (Suppl 2), 19644–19650. 10.1073/pnas.0901637106 19805041 PMC2780935

[B204] Soberón J. (2007). Grinnellian and Eltonian niches and geographic distributions of species. Ecol. Lett. 10, 1115–1123. 10.1111/j.1461-0248.2007.01107.x 17850335

[B205] Soberón J. M. (2010). Niche and area of distribution modeling: a population ecology perspective. Ecography 33, 159–167. 10.1111/j.1600-0587.2009.06074.x

[B206] Soininen J. (2012). Macroecology of unicellular organisms–patterns and processes. Environ. Microbiol. Rep. 4, 10–22. 10.1111/j.1758-2229.2011.00308.x 23757224

[B207] Song H.-S. Cannon W. R. Beliaev A. S. Konopka A. (2014). Mathematical modeling of microbial community dynamics: a methodological review. Processes 2, 711–752. 10.3390/pr2040711

[B208] Speth D. R. In ‘t Zandt M. H. Guerrero-Cruz S. Dutilh B. E. Jetten M. S. M. (2016). Genome-based microbial ecology of anammox granules in a full-scale wastewater treatment system. Nat. Commun. 7, 11172. 10.1038/ncomms11172 27029554 PMC4821891

[B209] Staniczenko P. P. A. Sivasubramaniam P. Suttle K. B. Pearson R. G. (2017). Linking macroecology and community ecology: refining predictions of species distributions using biotic interaction networks. Ecol. Lett. 20, 693–707. 10.1111/ele.12770 28429842 PMC5485222

[B210] Stocker T. F. Dahe Q. Plattner G.-K. (2014). Climate Change 2013: The Physical Science Basis: Working Group I Contribution to the Fifth Assessment Report of the Intergovernmental Panel on Climate Change (United Kingdom: Cambridge University Press).

[B211] Stocker T. (2014). Climate change 2013: the physical science basis: Working Group I contribution to the Fifth assessment report of the Intergovernmental Panel on Climate Change. (United Kingdom: Cambridge University Press).

[B212] Stocker E. F. Alquaied F. Bilanow S. Ji Y. Jones L. (2018). TRMM Version 8 Reprocessing Improvements and Incorporation into the GPM Data Suite. J. Atmos. Ocean. Technol. 35, 1181–1199. 10.1175/JTECH-D-17-0166.1

[B213] Succurro A. Ebenhöh O. (2018). Review and perspective on mathematical modeling of microbial ecosystems. Biochem. Soc Trans. 46, 403–412. 10.1042/BST20170265 29540507 PMC5906705

[B214] Sugimoto M. Kikuchi S. Tomita M. (2005). Reverse engineering of biochemical equations from time-course data by means of genetic programming. Biosystems 80, 155–164. 10.1111/j.1461-0248.2004.00701.x 15823414

[B215] Taudiere A. Violle C. (2016). cati: an R package using functional traits to detect and quantify multi-level community assembly processes. Ecography 39, 699–708. 10.1111/ecog.01433

[B216] Turner D. P. Ritts W. D. Cohen W. B. Gower S. T. Running S. W. Zhao M. (2006). Evaluation of MODIS NPP and GPP products across multiple biomes. Remote Sens. Environ. 102, 282–292. 10.1016/j.rse.2006.02.017

[B217] Ugarte A. Vicedomini R. Bernardes J. Carbone A. (2018). A multi-source domain annotation pipeline for quantitative metagenomic and metatranscriptomic functional profiling. Microbiome 6 (1), 1–27. 10.1186/s40168-018-0532-2 30153857 PMC6114274

[B218] Uusitalo L. (2007). Advantages and challenges of Bayesian networks in environmental modelling. Ecol. Modell. 203, 312–318. 10.1016/j.ecolmodel.2006.11.033

[B219] Vázquez-Castellanos J. F. Serrano-Villar S. Latorre A. Artacho A. Ferrús M. L. Madrid N. (2015). Altered metabolism of gut microbiota contributes to chronic immune activation in HIV-infected individuals. Mucosal Immunol. 8, 760–772. 10.1038/mi.2014.107 25407519

[B220] Veiga R. V. Barbosa H. J. C. Bernardino H. S. Freitas J. M. Feitosa C. A. Matos S. M. A. (2018). Multiobjective grammar-based genetic programming applied to the study of asthma and allergy epidemiology. BMC Bioinf. 19, 245. 10.1186/s12859-018-2233-z PMC6047363 29940834

[B221] Vieira-Silva S. Falony G. Darzi Y. Lima-Mendez G. Garcia Yunta R. Okuda S. (2016). Species–function relationships shape ecological properties of the human gut microbiome. Nat. Microbiol. 1, 16088. 10.1038/nmicrobiol.2016.88 27573110

[B222] von Meijenfeldt F. A. B. Arkhipova K. Cambuy D. D. Coutinho F. H. Dutilh B. E. (2019). Robust taxonomic classification of uncharted microbial sequences and bins with CAT and BAT. bioRxiv 530188, 1–14. 10.1101/530188 PMC6805573 31640809

[B223] Wan Z. Hook S. Hulley G. (2015). MOD11B3 MODIS/Terra Land Surface Temperature/Emissivity Monthly L3 Global 6km SIN Grid V006 [Data set]. NASA EOSDIS LP DAAC. 10.5067/MODIS/MOD11B3.006

[B224] Webb C. O. Ackerly D. D. McPeek M. A. Donoghue M. J. (2002). Phylogenies and Community Ecology. Annu. Rev. Ecol. Syst. 33, 475–505. 10.1146/annurev.ecolsys.33.010802.150448

[B225] Webb C. O. Ackerly D. D. Kembel S. W. (2008). Phylocom: software for the analysis of phylogenetic community structure and trait evolution. Bioinformatics 24, 2098–2100. 10.1093/bioinformatics/btn358 18678590

[B226] Wei C. L. Rowe G. T. Escobar-Briones E. Boetius A. Soltwedel T. Caley M. J. Pitcher C. R. (2010). Global patterns and predictions of sea- floor biomass using random forests. PloS One 5 (12), e15323. 21209928 10.1371/journal.pone.0015323 PMC3012679

[B227] Weiss S. Van Treuren W. Lozupone C. Faust K. Friedman J. Deng Y. (2016). Correlation detection strategies in microbial data sets vary widely in sensitivity and precision. ISME J. 10, 1669–1681. 10.1038/ismej.2015.235 26905627 PMC4918442

[B228] Widder S. Allen R. J. Pfeiffer T. Curtis T. P. Wiuf C. Sloan W. T. (2016). Challenges in microbial ecology: building predictive understanding of community function and dynamics. ISME J. 10, 2557–2568. 10.1038/ismej.2016.45 27022995 PMC5113837

[B229] Wilheit T. T. Chang A. T. C. Chiu L. S. (1991). Retrieval of Monthly Rainfall Indices from Microwave Radiometric Measurements Using Probability Distribution Functions. J. Atmos. Ocean. Technol. 8, 118–136. 10.1175/1520-0426(1991)008<0118:ROMRIF>2.0.CO;2

[B230] Willig M. R. Kaufman D. M. Stevens R. D. (2003). Latitudinal Gradients of Biodiversity: Pattern, Process, Scale, and Synthesis. Annu. Rev. Ecol. Evol. Syst. 34, 273–309. 10.1146/annurev.ecolsys.34.012103.144032

[B231] Wisz M. S. Pottier J. Kissling W. D. Pellissier L. Lenoir J. Damgaard C. F. (2013). The role of biotic interactions in shaping distributions and realised assemblages of species: implications for species distribution modelling. Biol. Rev. Camb. Philos. Soc 88, 15–30. 10.1111/j.1469-185X.2012.00235.x 22686347 PMC3561684

[B232] Wu J. Shen W. Sun W. Tueller P. T. (2002). Empirical patterns of the effects of changing scale on landscape metrics. Landsc. Ecol. 17, 761–782. 10.1023/A:1022995922992

[B233] Wu Y.-W. Simmons B. A. Singer S. W. (2016). MaxBin 2.0: an automated binning algorithm to recover genomes from multiple metagenomic datasets. Bioinformatics 32, 605–607. 10.1093/bioinformatics/btv638 26515820

[B234] Xiong X. Barnet C. Maddy E. Sweeney C. Liu X. Zhou L. (2008). Characterization and validation of methane products from the Atmospheric Infrared Sounder (AIRS). J. Geophys. Res. 113, 253. 10.1029/2007JG000500

[B235] Xue P.-P. Carrillo Y. Pino V. Minasny B. McBratney A. B. (2018). Soil Properties Drive Microbial Community Structure in a Large Scale Transect in South Eastern Australia. Sci. Rep. 8, 11725. 10.1038/s41598-018-30005-8 30082740 PMC6078944

[B236] Yilmaz P. Kottmann R. Field D. Knight R. Cole J. R. Amaral-Zettler L. (2011). Minimum information about a marker gene sequence (MIMARKS) and minimum information about any (x) sequence (MIxS) specifications. Nat. Biotechnol. 29, 415–420. 10.1038/nbt.1823 21552244 PMC3367316

[B237] Zhou J. Deng Y. Shen L. Wen C. Yan Q. Ning D. (2016). Temperature mediates continental-scale diversity of microbes in forest soils. Nat. Commun. 7, 12083. 10.1038/ncomms12083 27377774 PMC4935970

